# Producing high-quantity and high-quality recombinant adeno-associated virus by low-cis triple transfection

**DOI:** 10.1016/j.omtm.2024.101230

**Published:** 2024-03-12

**Authors:** Hao Liu, Yue Zhang, Mitchell Yip, Lingzhi Ren, Jialing Liang, Xiupeng Chen, Nan Liu, Ailing Du, Jiaming Wang, Hao Chang, Hyejin Oh, Chen Zhou, Ruxiao Xing, Mengyao Xu, Peiyi Guo, Dominic Gessler, Jun Xie, Phillip W.L. Tai, Guangping Gao, Dan Wang

**Affiliations:** 1 Horae Gene Therapy Center, University of Massachusetts Chan Medical School, Worcester, MA 01605, USA; 2 Department of Microbiology and Physiological Systems, University of Massachusetts Chan Medical School, Worcester, MA 01605, USA; 3 RNA Therapeutics Institute, University of Massachusetts Chan Medical School, Worcester, MA 01605, USA

**Keywords:** rAAV, biomanufacturing, triple transfection, plasmid backbone, potency

## Abstract

Recombinant adeno-associated virus (rAAV)-based gene therapy is entering clinical and commercial stages at an unprecedented pace. Triple transfection of HEK293 cells is currently the most widely used platform for rAAV manufacturing. Here, we develop low-cis triple transfection that decreases transgene plasmid use by 10- to 100-fold and overcomes several major limitations associated with standard triple transfection. This new method improves packaging of yield-inhibiting transgenes by up to 10-fold, and generates rAAV batches with reduced plasmid backbone contamination that otherwise cannot be eliminated in downstream processing. When tested in mice and compared with rAAV produced by standard triple transfection, low-cis rAAV shows comparable or superior potency and results in diminished plasmid backbone DNA and RNA persistence in tissue. Mechanistically, low-cis triple transfection relies on the extensive replication of transgene cassette (i.e., inverted terminal repeat-flanked vector DNA) in HEK293 cells during production phase. This cost-effective method can be easily implemented and is widely applicable to producing rAAV of high quantity, purity, and potency.

## Graphical abstract

**Figure:**
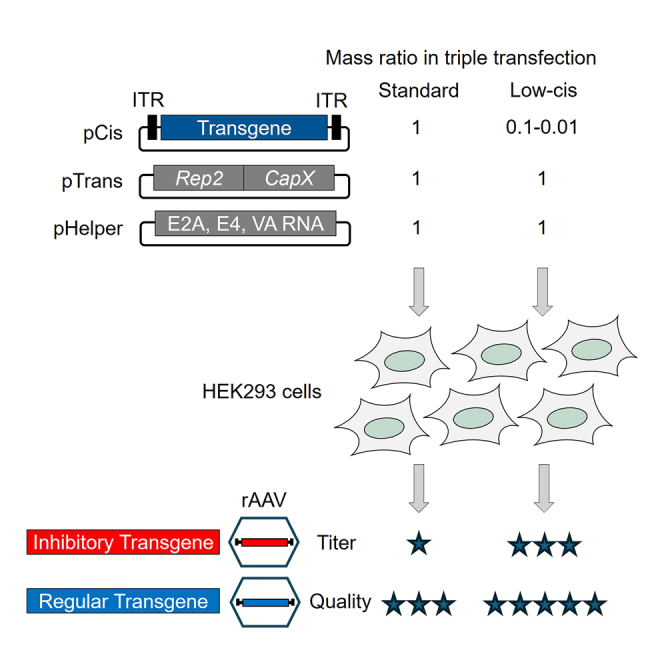

## Teaser

Liu and colleagues present a broadly applicable and cost-effective strategy for rAAV production. By dramatically cutting the pCis plasmid input, this low-cis triple transfection method significantly improves the titer of rAAV expressing inhibitory transgenes and decreases the rAAV impurity of plasmid backbone encapsidation.

## Introduction

**Figure 1:**
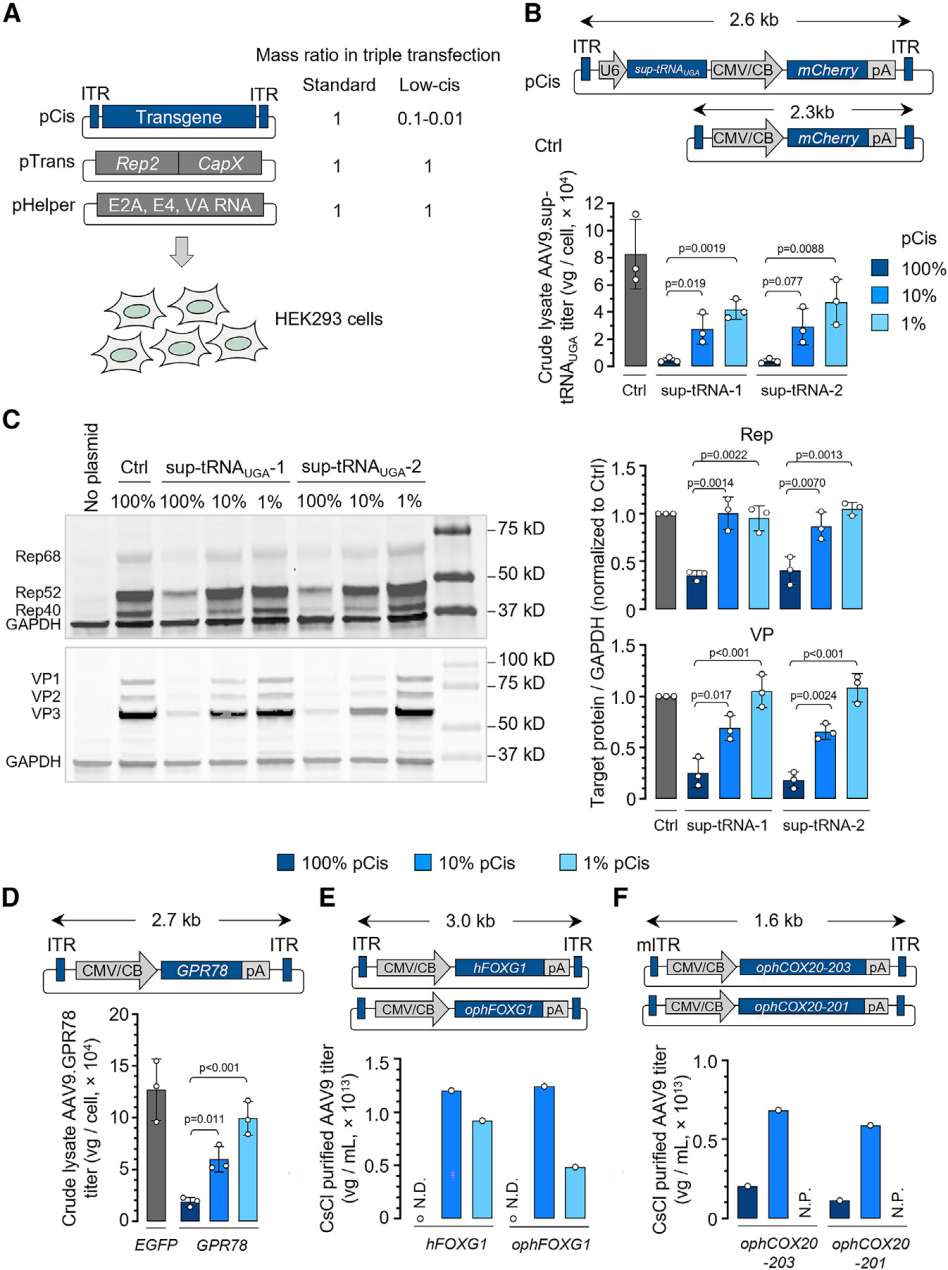
Low-cis triple transfection rescues AAV packaging of transgene cassettes that are incompatible with standard triple transfection (A) Schematic diagram comparing plasmid use between standard triple transfection and low-cis triple transfection. In standard triple transfection, roughly equal mass or mole of pCis, pTrans and pHelper plasmids are co-transfected to HEK293 cells to produce rAAV. This study adopted equal mass in standard triple transfection. In low-cis triple transfection, pCis is reduced to 10% to 1% of the amount used in standard triple transfection. Detailed plasmid use at various production scales is shown in Table S1. (B) (Top) Vector genome structures of pCis that expresses *sup-tRNA*_*UGA*_ and control pCis without *sup-tRNA*_*UGA*_. (Bottom) Packaging yield of ssAAV9.*sup-tRNA*_*UGA*_ using standard amount of pCis (100%) or reduced amount (10% or 1%). Sup-tRNA-1 and sup-tRNA-2 denote two different sup-tRNAs. In small-scale rAAV production, each well of adherent HEK293 cells in 12-well plate was transfected with certain plasmid amount as detailed in Table S1. Crude lysate was harvested 72 h after transfection followed by three successive freeze-thaw cycles. Cleared crude lysates after centrifugation were treated with DNase-I and protease K, followed by ddPCR to determine titer. (C) (Left) Western blotting of Rep proteins (top) and VPs (bottom) expression in transfected cells as shown in (B). (Right) Quantification of western blotting images. (D) (Top) Vector genome structure of pCis that expresses the *GPR78* transgene. (Bottom) Packaging yield of ssAAV9.GPR78 using various amounts of pCis, with an *EGFP* transgene construct packaged using 100% pCis as control. Small-scale rAAV production and titer determination procedures were the same as (B). (E) (Top) Vector genome structure of pCis that expresses human *FOXG1* (*hFOXG1*) or codon-optimized *hFOXG1* (*ophFOXG1*) transgene. (Bottom) Packaging yield of ssAAV9.hFOXG1 and ssAAV9.ophFOXG1 using standard or low-cis triple transfection. In large-scale rAAV production, 10 roller bottles of adherent HEK293 cells were transfected with certain plasmid amount as detailed in Table S1. Cells and culture media were harvested 72 h after transfection followed by CsCl density gradient ultracentrifugation. Purified rAAV was treated with DNase-I and protease K, followed by ddPCR to determine titer. N.D., no detectable titer. (F) (Top) Vector genome structures of pCis that expresses codon-optimized human COX20 isoform 203 (*ophCOX20-203*) or *ophCOX20-201* transgene. (Bottom) Packaging yield of scAAV9.ophCOX20-203 and scAAV9.ophCOX20-201 using 100% or 10% pCis input. N.P., not performed. The large-scale rAAV production, purification and titer determination procedures were same as (E). mITR, mutant ITR for generating scAAV. In (B–D), data are mean ± SD of three biological replicates. Statistical analysis was performed using one-way ANOVA followed by Dunnett’s multiple comparisons test against the 100% pCis group.

Gene therapy is revolutionizing the treatment of human diseases, especially rare genetic diseases. Currently, there are more than 200 clinical trials using recombinant adeno-associated virus (rAAV) as the gene therapy vector, creating a huge demand in rAAV manufacturing. Triple transfection is the most widely used method for producing rAAV.^1^ It involves three plasmids co-transfected into HEK293 cells at roughly equal molar or mass ratio: a helper plasmid that delivers adenovirus helper genes (pHelper), a *trans*-complementing plasmid that expresses AAV *Rep* and *Cap* genes (pTrans), and a cis plasmid that harbors a therapeutic transgene cassette flanked by AAV inverted terminal repeats (ITRs) (pCis) (Figure 1A). Although triple transfection has gained tremendous success, emerging issues arise when it is increasingly used to produce clinical-grade rAAV and scrutinized by advanced analytical assays, such as the high cost of goods for current good manufacturing practice (cGMP) plasmid,^2^ low yield in packaging certain transgenes,^3^^,^^4^ and alarming levels of encapsidated plasmid backbone DNA that raise safety concerns.^5^^,^^6^^,^^7^^,^^8^ As rAAV gene therapy is entering the clinic with an unprecedented pace,^9^ how to cost effectively manufacture rAAV at scale, and improve rAAV vector genome purity and potency, poses significant challenges to advancing gene therapy.

Since the advent of triple transfection,^1^ several groups have reported systematic optimization of the plasmid ratio and/or amount to improve rAAV yield.^10^^,^^11^ Notably, such optimization experiments routinely exploit a range of plasmid use of a 2- to 3-fold difference without mechanistic modeling. In addition, substantial endeavors have been made to improve the process, yield, or product purity. To decrease plasmid use and hence the cost of goods, dual-plasmid transfection was devised to eliminate pTrans by including *Rep/Cap* in pHelper or pCis.^12^^,^^13^ For packaging transgenes whose protein products are toxic to HEK293 cells and inhibitory to rAAV production, chemical-regulated riboswitch^14^ or RNA interference strategies^3^ are used to dampen the cytotoxicity caused by transgene expression during rAAV production. To decrease plasmid backbone encapsidation, minicircle plasmid or doggybone DNA that are devoid of bacterial backbone sequences have been used to provide an ITR-flanked transgene cassette instead of pCis.^15^^,^^16^ Nevertheless, these methods require sophisticated plasmid reconstruction and/or expertise to manufacture special raw DNA materials that are not readily available to common laboratories. The difficulty in combining these methods into a single system for multifaceted improvement has also limited their broad adoption. In addition, the prokaryotic DNA elements conferring transgene expression regulation may cause adverse immune response *in vivo*, posing a safety concern in human gene therapy applications.

Here, we developed a broadly applicable and cost-effective triple transfection method—low-cis triple transfection (Figures 1A; Table S1)—that can be easily implemented using standard plasmids and confers multiple advantages. By reducing the pCis amount to 10% or 1%, which is beyond the scope of transfection optimization, low-cis triple transfection greatly diminishes transgene expression and related cytotoxicity in HEK293 cells, thereby rescuing AAV packaging of yield-inhibiting transgenes driven by either a Pol II or Pol III promoter. In addition to packaging various inhibitory or permissive transgenes, low-cis rAAV encapsidates 2- to 10-fold less plasmid backbone DNA. Mechanistically, the transgene cassette in pCis undergoes rescue by replication in the production phase, and low-cis triple transfection enables more replication cycles that generate more full-length vector DNA to compensate for the low pCis input; concomitantly, the backbone-containing DNA replication product is diluted. Compared with rAAV produced by standard triple transfection, low-cis rAAV shows comparable or superior vector potency in a transgene-, serotype-, and tissue-specific manner following *in vivo* administration. Importantly, low-cis rAAV treatment is associated with much fewer plasmid backbone DNA and transcripts in tissue, an important safety consideration for human gene therapy applications.

## Results

### Low-cis triple transfection improves the yield of AAV vectors that are incompatible with standard triple transfection

We recently combined rAAV-based gene delivery and UAG-targeting suppressor tRNA-mediated stop codon readthrough to develop AAV-NoSTOP,^17^ an *in vivo* gene therapy approach that targets pathogenic nonsense mutations. As we attempted to expand the AAV-NoSTOP toolbox, we failed to package UGA-targeting suppressor tRNA (sup-tRNA_UGA_) genes into rAAV by standard triple transfection (Figure 1B). Mechanistically, we found that standard (i.e., 100%) pCis expressing sup-tRNA_UGA_ diminished Rep and Cap expression in HEK293 cells (Figure 1C). We hypothesized that reducing pCis can lower sup-tRNA_UGA_ expression, and therefore mitigate its inhibitory effects on Rep and Cap expression. Importantly, a small amount of pCis was routinely used to generate rAAV libraries to minimize cross-packaging, and the resulting rAAV titers were adequate.^18^^,^^19^ Strikingly, when we reduced pCis to 10% or 1%, the yield of single-stranded (ss) AAV9.sup-tRNA_UGA_ was increased by around 10-fold, reaching up to 50% of the control construct (Figure 1B); Rep and Cap expression was restored as expected (Figure 1C). The AAV9.sup-tRNA_UGA_ produced by low-cis triple transfection was functional in mediating UGA readthrough in HEK293 cells (Figure S1).

Next, we extended the low-cis method to package protein-coding genes driven by a Pol II promoter. We first tested packaging *GPR78,* a transgene known to inhibit rAAV production due to the cytostatic effect of its protein product G protein-coupled receptor 78.^3^ Consistent with a previous report,^3^ we found that the titer of AAV9.GPR78 was much lower than that of a control EGFP vector when using standard triple transfection (i.e., 100% pCis input) (Figure 1D). When pCis was reduced to 10% or 1%, rAAV yield was increased by 3- or 5-fold, respectively, reaching up to 80% of the control construct (Figure 1D). Prompted by this finding, we adapted low-cis triple transfection to produce therapeutic AAV vectors used in two ongoing gene therapy studies, because these vectors showed no or poor yield by standard triple transfection, despite multiple attempts. *FOXG1* encodes a transcription factor that is critical in neuron differentiation. Mutation in *FOXG1* causes movement disorder and seizure in FOXG1 syndrome patients.^20^ Initially, we attempted in vain to package human *FOXG1* (*hFOXG1*) or codon-optimized *hFOXG1* in AAV9 by standard triple transfection; by contrast, low-cis triple transfection generated robust rAAV9 titers that reached the normal range of 1 × 10^13^ vector genomes (vg)/mL (Figure 1E). In another case, we packaged two cDNA isoforms of *COX20*, deficiency of which causes ataxia and muscle hypotonia in children.^21^ Reducing the pCis to 10% increased the self complementary (sc) AAV9 vector yield by 3- to 6-fold (Figure 1F). Together, these results suggested that low-cis triple transfection can be broadly applicable to packaging yield-inhibiting transgenes that are incompatible with standard triple transfection.

### Application of low-cis triple transfection to packaging permissive transgenes

**Figure 2:**
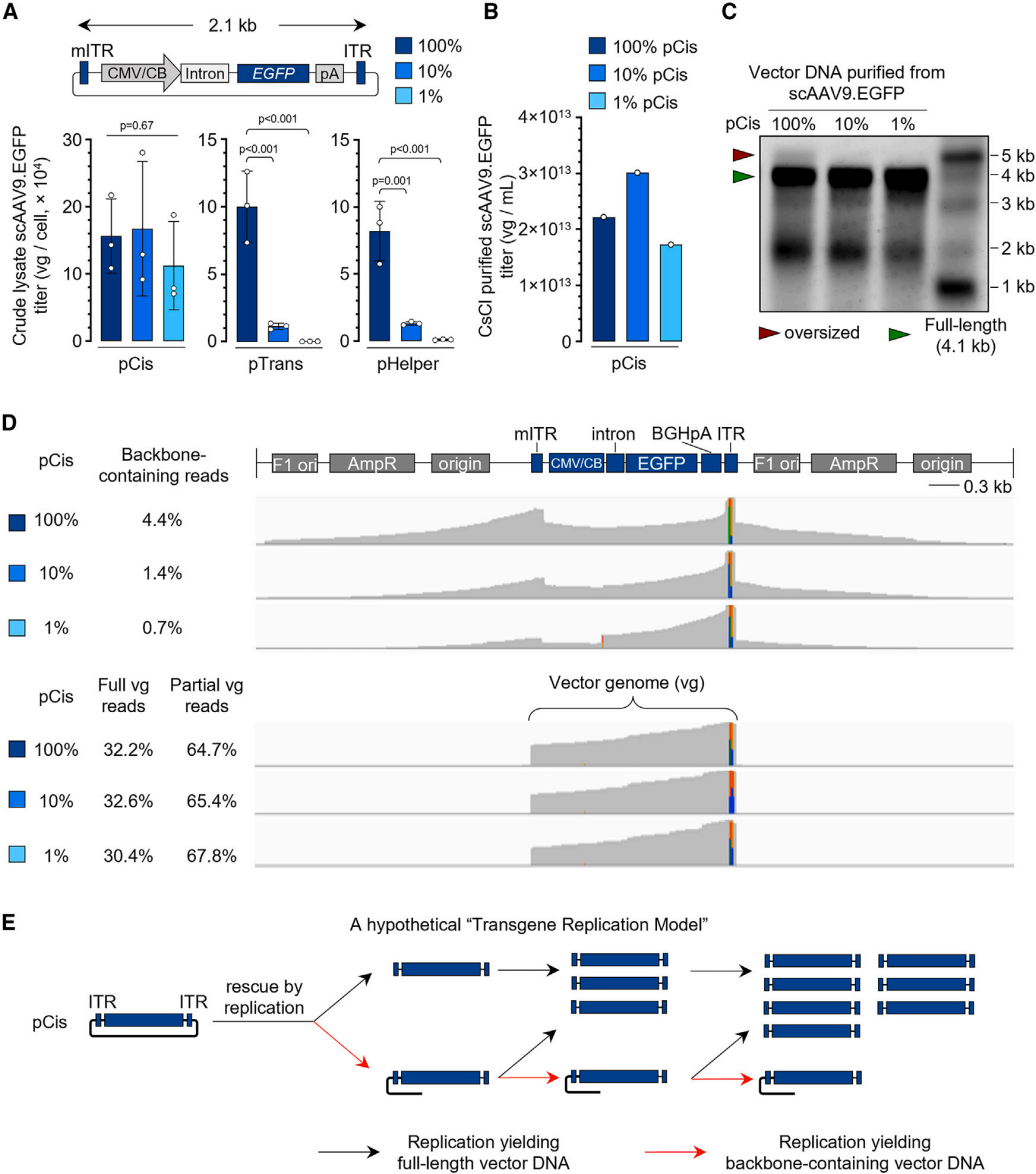
Low-cis triple transfection produces high-quality AAV vectors with reduced backbone DNA encapsidation (A) (Top) Vector genome structure of pCis that expresses *EGFP* transgene. (Bottom) Packaging yield of scAAV9.*EGFP* using various amounts of pCis (left), pTrans (middle), or pHelper (right) in small-scale rAAV production. (B) Packaging yield of scAAV9.*EGFP* using standard or low-cis triple transfection in large-scale rAAV production. (C) Denaturing alkaline gel image showing the size of vector DNA purified from scAAV9.EGFP in (B). Green arrowhead indicates the full-length vector genome size; red arrowhead indicates oversized vector genome, presumably due to the presence of plasmid backbone DNA. (D) PacBio sequencing analysis of vector DNA purified from scAAV9.EGFP as described in (B). (Top) Mapping of backbone-containing reads. The gray area indicates the abundance of backbone-containing reads mapped to the plasmid backbone of pCis reference shown on the top. The percentages of backbone-containing reads in scAAV9.EGFP produced using various pCis input are shown. (Bottom) Mapping of total reads. The gray area indicates the reads abundance mapped to the pCis reference shown on the top. The percentages of reads of full-length or partial vector genome (vg) size are shown. mITR: mutant ITR for generating scAAV. (E) A hypothetical transgene replication model showing how reducing pCis in triple transfection diminishes plasmid backbone DNA encapsidation. In (A), data are mean ± SD of three biological replicates. Statistical analysis was performed using one-way ANOVA followed by Dunnett’s multiple comparisons test against 100% pCis group.

Because low-cis triple transfection produced rAAVs bearing yield-inhibiting transgenes at high titers, we reasoned that it can be broadly applicable to packaging permissive transgenes. Using *EGFP* and Gaussia luciferase (*Gluc*) genes driven by a ubiquitous CMV/CB (CB6) or bi-directional promoter (BiP)^22^ as model constructs, we demonstrated that low-cis triple transfection was able to produce rAAVs of different transgenes, genome configurations (ss or sc), and capsids (AAV2, AAV5, or AAV9) (Figure 2A, S2A–C), although moderately reduced yield was observed in producing ssAAV9.EGFP and ssAAV5.Gluc-BiP-EGFP with 1% pCis input (Figures S2B and S2C). By stark contrast, when pTrans or pHelper was reduced to 10% or 1%, rAAV yield decreased in a linear manner (Figures 2A and S2D). These data support the notion that ITR-flanked vector DNA in pCis undergoes replication after transfection, and the replicated vector DNA, but not the DNA directly excised from pCis, is packaged into the AAV capsid.^23^^,^^24^^,^^25^^,^^26^ Consistent with a previous report,^27^ vector DNA replication from homogeneous pCis resulted in diversified ITR configuration in the packaged rAAV genome with the ratio fitting a mathematical replication model^27^ (Figure S3), lending further support to the rescue by replication model.^15^^,^^24^

### Low-cis triple transfection produces high-quality rAAV with reduced plasmid backbone encapsidation

To further characterize rAAVs produced by standard or low-cis triple transfection, we produced scAAV9.EGFP in large scale of 10 roller bottles of adherent HEK293 cells, and purified by cesium chloride density gradient ultracentrifugation (Table S1). The rAAV titers were largely comparable (Figure 2B). We then analyzed vector genome integrity by resolving DNA extracted from scAAV9.EGFP on a denaturing alkaline gel. Although the 4.1-kb band of the full-length genome was comparable between standard and low-cis rAAVs, we noticed that an oversized DNA species in standard rAAV, presumably representing vector DNA containing the plasmid backbone,^27^ was no longer visible in low-cis rAAV (Figure 2C). This observation indicated that low-cis triple transfection produced rAAV with diminished plasmid backbone DNA encapsidation. To quantitatively measure vector genome identity, we performed PacBio long-read sequencing of purified scAAV9.EGFP vector DNA, and analyzed the reads mapped to the pCis backbone. The backbone-containing reads were reduced from 4.4% in standard triple transfection to 1.4% and 0.7% when pCis was reduced to 10% and 1%, respectively (Figure 2D). By contrast, the reads mapped to full-length or partial vector genome were comparable between standard and low-cis triple transfection (Figure 2D), consistent with the observation by alkaline gel electrophoresis (Figure 2C). The unbiased PacBio sequencing dataset also allowed us to interrogate other vector impurities derived from host cell genome and pHelper plasmid. We found that the total reads mapped to host cell genome and pHelper were comparable among rAAV preparations, although low-cis rAAV contained slightly reduced host cell genome, but increased pHelper sequences (Table S2). To quantify encapsidated pCis backbone DNA in rAAV produced under different conditions (scale, capsid, transgene, and genome configuration), we performed a duplexing droplet digital PCR (ddPCR) assay following a stringent DNase I treatment protocol (Figures S4A and S4B) and found that low-cis triple transfection consistently reduced plasmid backbone DNA encapsidation (Figure S4C), a major source of vector impurity that otherwise cannot be eliminated by downstream processing.

How does low-cis triple transfection reduce plasmid backbone encapsidation in rAAV? Here, we propose a hypothetical transgene replication model as the potential mechanism (Figure 2E). We postulate that, after transfection to HEK293 cells, the ITR-flanked transgene cassette in pCis plasmid is replicated to generate either full-length vector DNA that contains only ITR-flanked transgene or backbone-containing vector DNA that contains plasmid backbone due to ITR readthrough, both of which are subject to subsequent replication cycles. After each round of replication, the backbone-containing vector DNA generates either full-length or backbone-containing DNA, while full-length DNA only replicates itself. The more rounds of replication, the lower the ratio of backbone-containing vector DNA. Compared with standard triple transfection, more replication cycles occur in low-cis triple transfection, which not only achieves comparable vector titer, but leads to greater vector DNA purity with reduced pCis backbone encapsidation (Figure 2E).

### *In vivo* potency of low-cis rAAV

**Figure 3:**
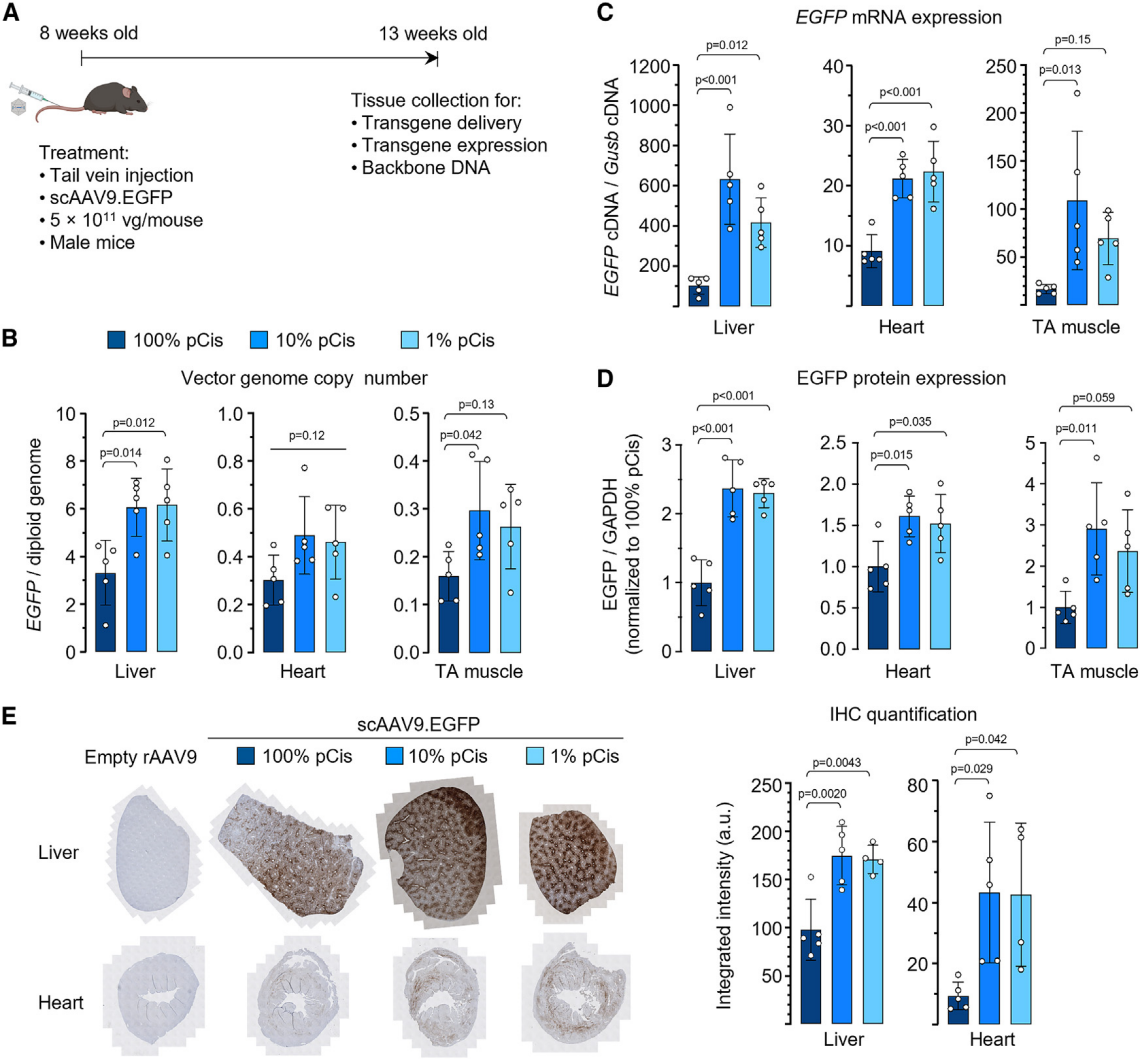
*In vivo* characterization of AAV vector potency (A) Schematic diagram showing workflow. (B–D) Quantification of *EGFP* DNA (B), cDNA (C), and protein (D) abundance in the liver, heart, and TA muscle from mice treated with scAAV9.EGFP vectors produced using different pCis input as described in Figure 2B. The original western blotting images for (D) are shown in Figure S5. (E) Representative EGFP IHC images (left) and quantification of IHC intensities (right) of liver and heart sections from mice as shown in (A). a.u., arbitrary unit. Note: one mouse sample in 1% pCis group was lost during IHC processing in the core. In (B–E), data are mean ± SD of individual animals (circles). Statistical analysis was performed using one-way ANOVA followed by Dunnett’s multiple comparisons test against 100% pCis group.

Next, we compared the *in vivo* potency of scAAV9.EGFP produced by standard or low-cis triple transfection following systemic injection to C57BL/6 mice (Figure 3A). Similar to previous observations that rAAV containing less pCis backbone DNA has higher potency,^15^ we found that low-cis rAAV showed higher gene delivery (Figure 3B) and transgene expression (Figures 3C–3E, S5) in the liver, heart, and tibialis anterior (TA) skeletal muscle. However, in comparing ssAAV9.Gluc-BiP-EGFP and ssAAV5.Gluc-BiP-EGFP batches, low-cis rAAVs led to a mild increase in transgene expression in the liver and serum, but did not outperform standard rAAVs in the heart and TA muscle (Figures S6 and S7). Together, these *in vivo* studies showed that low-cis rAAV is at least as potent as standard rAAV, and may exhibit enhanced potency in a transgene-, serotype-, and tissue-dependent manner.

### *In vivo* administration of low-cis rAAV leads to diminished backbone DNA and RNA in tissue

**Figure 4:**
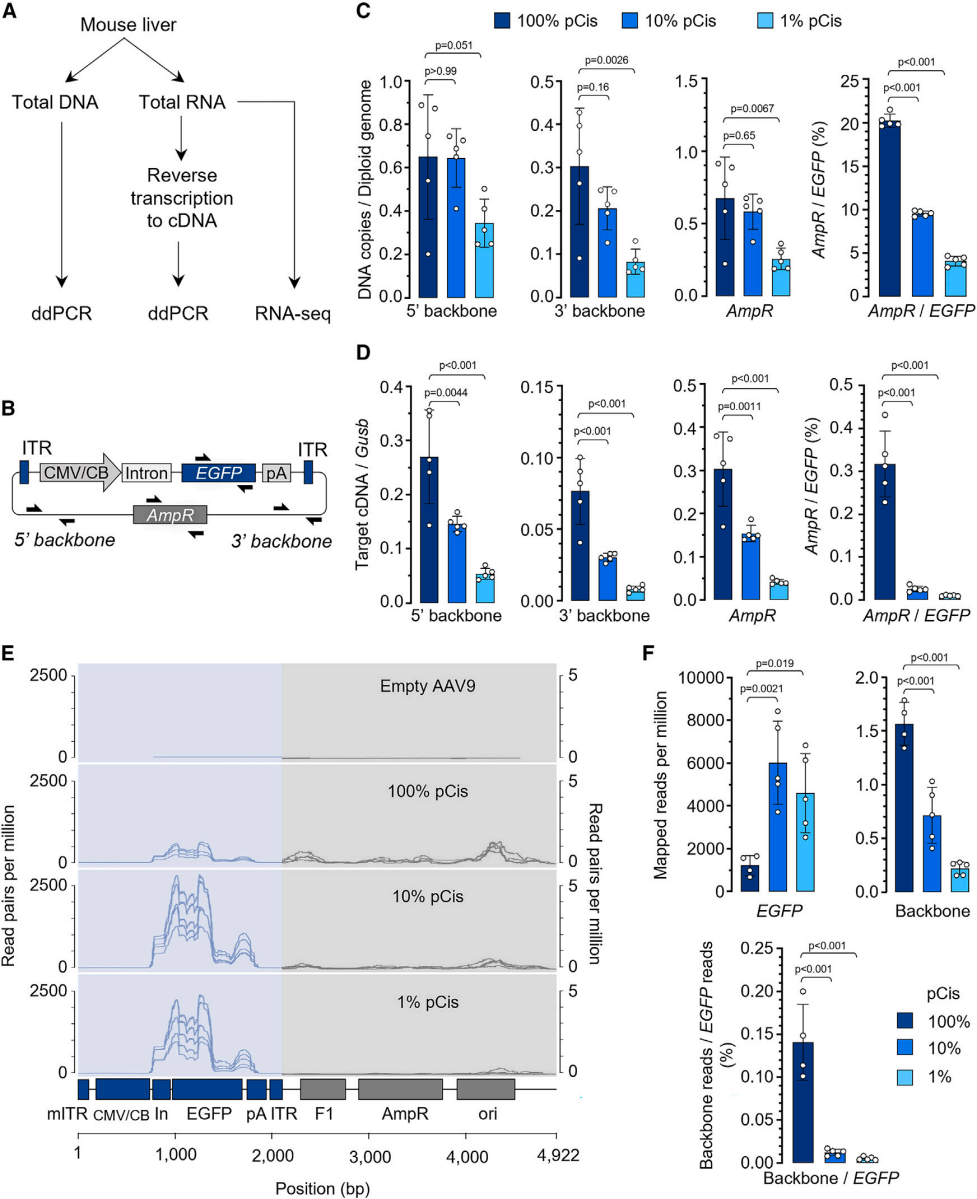
*In vivo* plasmid backbone DNA and RNA contaminants are significantly mitigated in AAV vectors produced by low-cis triple transfection (A) Schematic diagram showing the workflow of quantifying plasmid backbone DNA and RNA in mouse liver following scAAV9.EGFP treatment as described in Figure 3A. (B) Schematic of the pCis showing Taqman reagent designs that target the *EGFP* transgene, 5′ backbone, ampicillin resistant gene (*AmpR*), or 3′ backbone. (C and D) Quantification of plasmid backbone DNA (C) and cDNA (D) in mouse liver as described in Figure 3A. (E) Coverage plot of RNA-seq reads mapped to the scAAV9.EGFP vector genome flanked by ITRs (blue) or pCis backbone DNA (gray). mITR, mutant ITR for generating scAAV. (F) Quantification of the RNA-seq reads mapped to *EGFP* (top left), pCis backbone (top right), and their ratio (bottom). Note: one mouse sample in the 100% pCis group was deemed outlier due to very low overall mapped RNA reads, and therefore excluded from the RNA-seq analysis (see details in Figure S8). In (C), (D), and (F), data are mean ± SD of individual animals (circles). Statistical analysis was performed using one-way ANOVA followed by Dunnett’s multiple comparisons test against 100% pCis group.

The presence of plasmid backbone DNA sequences and their expression in tissues is an important safety concern of rAAV gene therapy.^5^^,^^6^ Similar to a previous finding,^28^ high levels of plasmid backbone DNA were detected in the mouse liver following rAAV treatment, and the relative abundance of ampicillin resistance gene (*AmpR*) to *EGFP* transgene reached 20% when rAAV was produced by standard triple transfection (Figures 4A–4C). Treatment with low-cis rAAV resulted in 2- to 4-fold less plasmid backbone DNA in the liver (Figure 4C). In addition, we found that backbone-derived RNA transcripts reached up to 30% of the house-keeping gene *Gusb* by rAAV produced from standard triple transfection, whereas low-cis rAAV reduced the RNA contaminants by 2- to 6-fold (Figure 4D). When normalized to the transgene *EGFP* transcripts, the backbone to *EGFP* ratio was reduced by 11- to 39-fold (Figure 4D). To comprehensively characterize backbone-derived RNA species *in vivo*, we performed RNA sequencing (RNA-seq) and mapped the reads to the pCis reference sequence. Interestingly, we found that most backbone transcripts were mapped to the ITR-adjacent regions (Figures 4E and S8), likely due to the inherent promoter activity of ITR.^29^^,^^30^ Consistent with the ddPCR assay results (Figure 4D), low-cis rAAV resulted in a reduction of backbone transcripts by 2- to 8-fold with a concomitant increase in *EGFP* transcripts up to 6-fold (Figure 4F); therefore, the undesired backbone transcripts, when normalized to that of *EGFP*, were reduced by 12- to 27-fold using low-cis rAAV (Figure 4F).

### Application of low-cis triple transfection in suspension HEK293 cells

**Figure 5:**
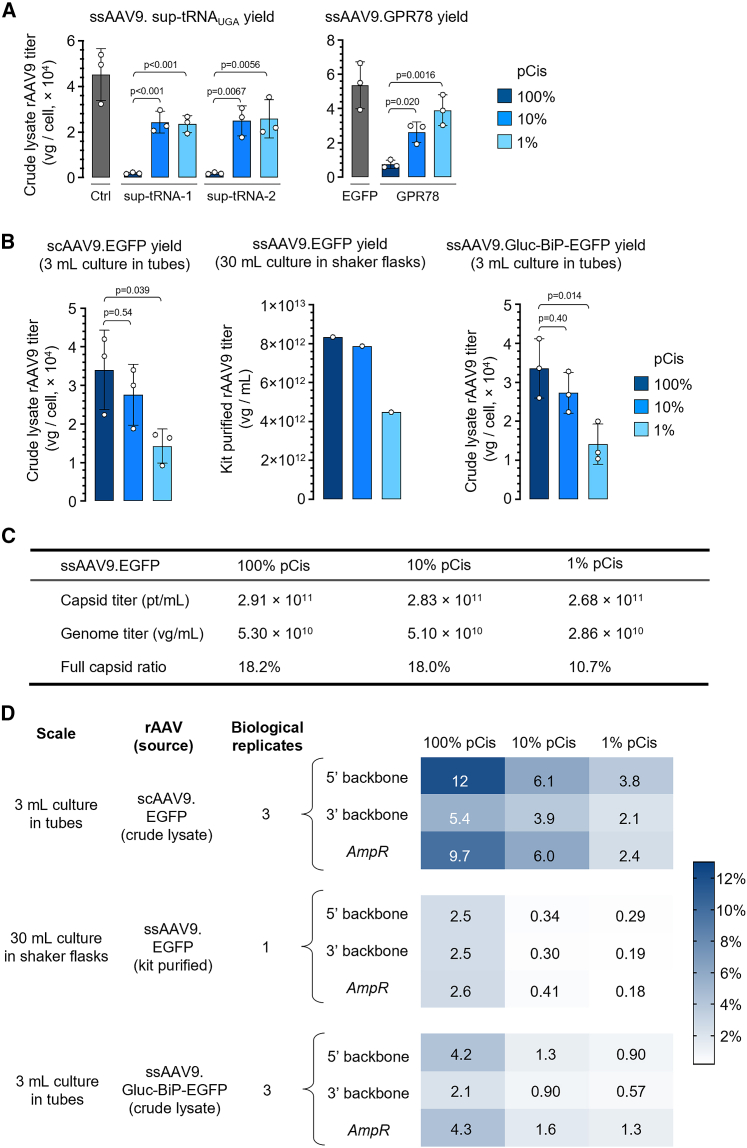
Application of low-cis triple transfection in suspension HEK293 cells (A) Packaging yield of ssAAV9.sup-tRNA_UGA_ (left) and ssAAV9.GPR78 (right) using standard amounts of pCis (100%) or reduced amount (10% or 1%) in 3 mL cell culture. The control construct (Ctrl) in the left panel is the same as the Ctrl in Figure 1B. In a 14-mL tube, 3 mL suspension HEK293 cells were transfected with certain amount of plasmids as detailed in Table S1. Crude lysate was harvested 72 h after transfection followed by three successive freeze-thaw cycles. Cleared crude lysates after centrifugation were treated with DNase-I and protease K, following by ddPCR to determine titer. (B) Packaging yield of scAAV9.EGFP in 3 mL cell culture (left), ssAAV9.EGFP in 30 mL of cell culture (middle), or ssAAV9.Gluc-BiP-EGFP in 3 mL cell culture (right). For rAAV production at the 30-mL scale, suspension HEK293 cells were transfected with certain plasmid amount as detailed in Table S1. Cells were pelleted 72 h after transfection and purified by a commercial kit as described in Methods. Purified rAAV were treated with DNase-I and protease K, followed by ddPCR to determine titer. (C) Tabulation of ssAAV9.EGFP capsid titer determined by ELISA and vector genome titer determined by ddPCR, and the calculated full capsid ratio in crude lysate of 30 mL cell culture. The full capsid ratio is calculated as genome titer divided by capsid titer. (D) Heatmap showing the percentage of pCis plasmid backbone DNA normalized to transgene in AAV9 vectors differing in production scale, source of material, transgene, and genome configuration as indicated. Cleared crude lysates or purified rAAV was treated with DNase-I and protease K, followed by duplex ddPCR with one probe targeting transgene, the other targeting 5′, 3′, or AmpR backbone as described in Figure 4B. For assays using multiple biological replicates, the average value was shown. In (A) and (B), data are mean ± SD of biological replicates. Statistical analysis was performed using one-way ANOVA followed by Dunnett’s multiple comparisons test against 100% pCis group.

Finally, we applied low-cis triple transfection to suspension HEK293 cells, a platform commonly used in rAAV manufacturing at scale, including at the cGMP level. Consistent with the results obtained using adherent HEK293 cells, low-cis triple transfection of suspension HEK293 cells enabled packaging the yield-inhibiting transgenes of *sup-tRNA*_*UGA*_ and *GPR78* (Figure 5A). When packaging an *EGFP* transgene, 10% pCis and standard triple transfection resulted in comparable production yield and full particle ratio, whereas using 1% pCis input was associated with a 50% decrease in yield and less full particles (Figures 5B and 5C), suggesting that the extent of pCis reduction can be optimized. Similar to using adherent HEK293 cells, the encapsidated plasmid backbone DNA was reduced by 2- to 10-fold when using low-cis triple transfection in suspension cell culture (Figure 5D).

## Discussion

The low-cis triple transfection method is easy to implement, cost effective, and able to produce rAAV of high quantity, purity, and potency. It leverages the extensive replication of ITR-flanked vector DNA in HEK293 cells to dramatically decrease pCis input and diminish backbone DNA encapsidation concomitant with vector DNA replication. We note that pCis reduction by up to 99% is fundamentally distinct from transfection optimization that operates within a narrower range of plasmid use.^10^^,^^11^ The large extent of pCis reduction enables packaging inhibitory transgenes and reducing plasmid backbone encapsidation.

When packaging yield-inhibiting transgenes, several advantages set our low-cis triple transfection method apart from other strategies, such as small hairpin RNA-mediated silencing of transgene expression,^3^ a combination of engineered cell line and regulatable promoter,^31^ or using insect cells where mammalian promoters are inactive.^4^ First, there is no need to modify pCis or introduce regulatory components that are therapeutically irrelevant. Second, low-cis triple transfection is applicable to transgenes driven by any promoters, including Pol III promoters, such as a *sup-tRNA* transgene. Third, rAAVs produced in HEK293 cells exhibit better purity and potency than those produced in insect cells.^32^^,^^33^

Beside reducing plasmid costs, another major advantage of low-cis triple transfection is the improved rAAV genome purity. Here, we propose the transgene replication model that transgene cassette replication attenuates plasmid backbone ratio in packaged rAAV (Figure 2E). This model is supported by the findings that (1) ITR configuration is homogeneous in pCis, but greatly diversified in rAAV caused by DNA replication during production (Figure S3); and (2) plasmid backbone encapsidation continuously decreases when pCis is reduced from 100% to 10%–1% (Figures 2D, 5D, and S4C). We postulate that more rounds of replication cycles occur when the pCis amount is reduced, thus causing diminished plasmid backbone encapsidation. Similar findings were reported in a recent study using seed rAAV instead of pCis to provide an ITR-flanked transgene cassette for rAAV production.^34^ The seed rAAV was produced by standard triple transfection and contained 2.8% plasmid backbone DNA. Interestingly, when the seed rAAV was used at a multiplicity of infection (MOI) of 1,500, plasmid backbone DNA in the output rAAV batch decreased to 0.3% owing to vector genome replication in the production phase. Reducing seed rAAV input to a lower MOI of 50 further decreased the plasmid backbone DNA in the output rAAV to 0.05%, presumably due to more extensive vector DNA replication.

The transgene replication model also explains why reducing pCis, but not pTrans or pHelper, generates comparable rAAV titer as only pCis, or specifically the ITR-flanked transgene cassette in pCis, is amplified by replication during rAAV production phase. This notion is supported by the findings that a small amount of pCis is sufficient to generate AAV libraries of adequate titers.^18^^,^^19^ Provided that the initial pCis template input exceeds a “threshold,” more pCis input does not generate higher vector yield, but adversely results in a higher backbone ratio. Similar phenomena were observed in the above-mentioned study: when the MOI of seed rAAV input was increased from 50 to 100, 500, and 1,500, the output rAAV titer remained largely unchanged, but the plasmid backbone ratio gradually increased.^34^ It is noteworthy that 1% pCis occasionally led to suboptimal rAAV yield (Figures S2B and S2C) and a reduced full capsid ratio (Figure 5C). One possible reason is that, as the stoichiometric ratio of pCis to pHelper and pTrans drastically decreases, a greater proportion of HEK293 cells are only transfected with pHelper and pTrans, generating empty capsids. When implementing low-cis transfection, the extent of pCis reduction can be optimized for rAAV genome titer, as well as the full capsid ratio, because our results suggest that the optimal pCis amount is not a set value, but depends on rAAV production condition, such as transgene, serotype, scale, genome configuration, transfection reagent, and cell line.

Plasmid backbone DNA encapsidation is an important safety concern in AAV-mediated human gene therapy.^5^^,^^6^^,^^7^^,^^8^ Reduced plasmid backbone DNA in low-cis rAAV directly contributes to lower backbone DNA and backbone-derived RNA contaminants in tissues following *in vivo* administration. In addition, it may also contribute to greater *in vivo* potency, because backbone DNA is rich in microbial-derived hypomethylated CpG content, which may elicit innate immune response and interfere with therapeutic efficacy.^6^^,^^35^ Alternatively, a lower pCis input and more extensive vector genome replication may have an impact on the epigenetic modification of rAAV vector genome, which may lead to improved vector potency *in vivo*.

In summary, the low-cis triple transfection is simple, scalable, and cost effective. This methodology takes advantage of transgene cassette replication to produce high-quantity and high-quality AAV vectors, and may serve as an improved manufacturing solution for human gene therapy.

## Materials and methods

### Cell culture

Adherent HEK293 cells (CRL-1573) were purchased from ATCC and maintained in DMEM (Gibco, 11965-084) supplemented with 10% (v/v) fetal bovine serum (FBS) (Gibco, 26140-079) and 1% (v/v) penicillin/streptomycin (Thermo Fisher Scientific, 15140122) under 37°C with 5% CO_2_. Suspension Expi293F cells were purchased from Thermo Fisher Scientific (A14528) and maintained in Freestyle F17 media (Gibco, A13835) supplemented with 10 mM Glutamax (Gibco, 35050061) under 37°C with 5% CO_2_, 80% humidity, and 120 rpm shaking speed.

### AAV vector production using adherent HEK293 cells

Small-scale vector preparations were generated in 12-well plates by standard triple transfection of HEK293 cells with three plasmids carrying the vector genome (pCis), Rep/Cap (pTrans, pRep2/CapX; Addgene #112865), and adenovirus helper genes (pHelper; Addgene #112867), respectively, at equal mass ratio of 1:1:1 using the calcium phosphate method (Promega, E1200), totaling 1.5 μg/well. In low-cis triple transfection, the pCis plasmid was reduced to 10% or 1% by mass. Transfected cells were maintained in DMEM supplemented with 10% FBS and 1% penicillin/streptomycin for 24 h, after which the culture medium was replaced with DMEM supplemented with 1% penicillin/streptomycin without FBS. 72 h after transfection, cells and culture media were harvested, and subjected to three successive freeze-thaw cycles. The crude lysates were centrifuged at 14,000 g/min for 15 min at 4°C to remove cell debris. Cleared crude lysates were treated with DNase-I and protease K before tittering by ddPCR.

Large-scale AAV vectors were produced similarly by calcium phosphate method using 4.5 mg total plasmids and approximately 1 × 10^9^ cells cultured in 10 roller bottles. At 72 h after transfection, AAV vectors were purified by two rounds of cesium chloride density gradient ultracentrifugation and dialysis.^36^ Titers of purified AAV vectors were quantified by ddPCR. To generate empty particles, HEK293 cells were transfected with pHelper and pTrans (without pCis). At 72 h after transfection, rAAV particles were purified using cesium chloride density gradient ultracentrifugation and quantified by SDS-PAGE and SYPRO Ruby protein stain (Thermo Fisher Scientific, S12000).

### AAV vector production using suspension Expi293F cells

Small-scale vector preparations were generated in 3 mL of cells cultured in 14 mL sterile polypropylene culture tubes (Thermo Fisher Scientific, 14-956-1J) placed on a customized 3D printed rack at a density of 1 × 10^6^ cells/mL with a shaking speed of 320 rpm. We found that this culture condition is superior to using commercial 48-well deep well plates in terms of cell viability and vector yield. Expi293F cells (Thermo Scientific, A14528) were transfected with three plasmids carrying the vector genome (pCis), Rep/Cap (pTrans, pRep2/CapX), and adenovirus helper genes (pHelper) at equal mass ratio of 1:1:1 using PEI Max (PolySciences, 24765-2), totaling 3 μg. In low-cis triple transfection, the pCis plasmid was reduced to 10% or 1% by mass. Transfected cells were maintained in Freestyle F17 media supplemented with 10 mM Glutamax for 72 h before harvest. Harvested cells were subjected to three successive freeze-thaw cycles. The crude lysates were centrifuged at 14,000 g/min for 15 min at 4°C to remove cell debris. Cleared crude lysates were treated with DNase-I and protease K before tittering by ddPCR.

Medium-scale AAV vectors were produced similarly by PEI Max method in a 125-mL shaker flask containing 30 mL cultured cells. At 72 h after transfection, AAV vectors were purified by AAVpro Purification Kit (Takara Bio, 6675). Purified AAV vector titer was quantified by ddPCR.

### Quantification of plasmid backbone in AAV vector preparations

rAAV crude lysates or purified AAV vectors were treated with DNase-I (Roche Life Science, 4716728001) and protease K (QIAGEN, 19133), as described previously.^37^ Duplexing Taqman ddPCR assays were performed with one reagent targeting *EGFP* transgene (Thermo Fisher Scientific, Mr00660654_cn), and the other targeting plasmid backbone. The plasmid backbone ratio in vector DNA was calculated by normalizing the plasmid backbone concentration over that of *EGFP*.

The 5′ plasmid backbone Taqman reagent included the following: forward primer: 5′-CACTCATTAGGCACCCCAG; reverse primer: 5′-GTTATCCGCTCACAATTCCAC; probe: 5′-ACACTTTATGCTTCCGGCTCGTATGTT; 3′ plasmid backbone Taqman reagent: forward primer: 5′-GACCGCTACACTTGCCAG; reverse primer: 5′-CCCCGATTTAGAGCTTGACG; probe: 5′-CTTCCTTTCTCGCCACGTTCGC; *AmpR* plasmid backbone Taqman reagent: forward primer: 5′-GATAAATCTGGAGCCGGTGAG; reverse primer: 5′-AGATAACTACGATACGGGAGGG; probe: 5′-TGGGTCTCGCGGTATCATTGCAG.

### Quantification of rAAV full capsid ratio

rAAV genome titer (vg/mL) in crude lysate was quantified by ddPCR using a Taqman reagent targeting the *EGFP* transgene (Thermo Fisher Scientific, Mr00660654_cn) after treatment with DNase-I (Roche Life Science, 4716728001) and protease K (QIAGEN, 19133). rAAV particle concentration (pt/mL) in crude lysate was quantified using the AAV9 Xpress ELISA Kit (PROGEN Biotechnik, PRAAV9XP) following the manufacturer’s manual. The full capsid ratio is calculated as the genome titer divided by the capsid titer.

### Western blotting

Cultured cells were pelleted by centrifugation at 1,000 g/min for 5 min at 4°C, and then lysed with M-PER (Thermo Fisher Scientific, 78501) with protease inhibitor (Roche, 4693159001). Mouse tissues were homogenized by TissueLyser II (Qiagen) in ice-cold T-PER (Thermo Fisher Scientific, 78510) with protease inhibitor (Roche, 4693159001). The protein concentration was determined using Pierce BCA Protein Assay Kit (Pierce, 23225). Normalized protein lysates were boiled for 10 min in reducing SDS sample buffer (Boston BioProducts, BP-111R). Primary antibodies were as follows: mouse anti-Rep (Origen Technologies, AM09104PU-N, 1:100), mouse anti-VP1/2/3 (PROGEN Biotechnik, 61058, 1:200), mouse anti-EGFP (Abcam, AB184601, 1:2000), rabbit anti-GAPDH (Abcam, ab9485, 1:2000). Secondary antibodies we as follows: LICOR IRDye 680RD goat anti-mouse IgG (H + L) (LI-COR Biosciences, 926-68070, 1:3000), LICOR IRDye 800CW goat anti-rabbit IgG (H + L) (LI-COR Biosciences, 926-32211, 1:3000). Blot membranes were imaged by LI-COR scanner (Odyssey) and quantified by ImageJ Fiji.

### AAV vector DNA analysis by alkaline agarose gel electrophoresis

We prepared 0.8% agarose gel by boiling agarose in ultra-pure water, followed by cooling to 55°C and adding 0.1 volume of 10× alkaline gel electrophoresis buffer (500 mM NaOH and 10 mM EDTA). We treated 200 μL purified rAAV with DNase-I and protease K, and then purified by phenol:chloroform:isoamyl alcohol solution. Purified vector DNA was mixed with 6× alkaline gel loading buffer (Thermo Fisher Scientific, AAJ62157AB), and loaded to alkaline gel. Electrophoresis was performed at a voltage of 3V/cm for approximately 3 h. Then the gel was soaked in neutralization solution (BioWorld, 10750014) for 1 h at room temperature. The neutralized gel was stained with SYBR Gold (1:10,000 dilution, Thermo Fisher Scientific, S-11494) in 1× TAE buffer for 15 min, and imaged using a Bio-Rad Gel Doc XR+ Imaging System.

### Animal use and treatment

We purchased 8-week-old male C57BL/6J mice from The Jackson Laboratory (Stock # 000664). AAV vectors were diluted in 300 μL of DPBS (Sigma-Aldrich, D8537-6X500ML), and injected to mice via tail vein at a dose of 5 × 10^11^ vg/mouse. At 5 weeks after injection, mice were sacrificed for tissue collection. All animal experiments were reviewed and approved by the Institutional Animal Care and Use Committee of University of Massachusetts Chan Medical School.

### Histology and immunohistochemistry

Mouse tissues were fixed in 10% formalin (Thermo Fisher Scientific, SF100-20) overnight and embedded in paraffin. Sectioning and immunohistochemistry (IHC) with rabbit anti-EGFP (Thermo Fisher Scientific, A-11122, 1:500) were performed at the Morphology Core of University of Massachusetts Chan Medical School. Images were acquired on TissueFAXS SL at the Microscopy Core of University of Massachusetts Chan Medical School. Quantification of EGFP IHC intensity was performed using ImageJ Fiji as previously described.^38^

### Quantification of EGFP-positive cells

Adherent HEK293 cells were detached by trypsin treatment and collected in 1.5-mL microcentrifuge tubes. Cells were pelleted by centrifugation, washed, and resuspended in 1× PBS (Corning, 21-031-CV). GFP-positive cells (in percentage of total cells) were measured using Cellometer K2 (Nexcelom Bioscience, GFP_Transfection Rate program).

### Luciferase activity assay

Gaussia luciferase (Gluc) activity in cell culture media or mouse serum was measured using the Pierce Gaussia Luciferase Flash Assay kit (Thermo Fisher Scientific, 16159) following the manufacturer’s instructions. Luminescence signal was detected using a plate reader (BioTek).

### PacBio sequencing and bioinformatic analysis

We treated 200 μL purified rAAV with DNase-I and proteinase K and then purified with a phenol:chloroform:isoamyl alcohol solution (25:24:1) (Thermo Fisher Scientific, 15593031). Samples were then subjected to ethanol precipitation and resuspended in nuclease-free water as described previously.^27^ Purified vector DNA was heated and strand annealed in annealing buffer (25 mM NaCl, 10 mM Tris-HCl, 0.5 mM EDTA) at 95°C for 5 min and then slowly cooled to 25°C (1 min for every 1°C) on a thermocycler. Lambda phage DNA digested with PstI (NEB, R0140) was used as spike-in for all libraries (10% by mass), serving as a normalizer for size loading bias. DNA samples were subjected to singe molecule, real-time (SMRT) sequencing at the Deep Sequencing Core of University of Massachusetts Chan Medical School. Vector DNA libraries were constructed using the Express Template Prep Kit 2.0 (PacBio, 100- 938-900) and ligated to indexed SMRTbell adapters with the Barcoded Overhang Adapter Kit (PacBio, 101-628-400/500). Pooled DNA libraries were purified using 1.8× AMPure beads. Sequencing was carried out on a Sequel II instrument on a single flow cell.

Raw subreads underwent preprocessing prior to bioinformatic analysis as described previously.^32^ Subreads were initially processed through recalladapters 9.0.0 with the following parameters: −minSnr = 2.0 and −disableAdapterCorrection. Following recalladapters, the consensus read fastq file was generated using the circular consensus sequencing (CCS) tool in SMRT Link (10.1.0.119588) using the following settings: –minSnr = 3.75, –minPasses = 2, −byStrand. Downstream analyses were performed on the Galaxy web platform.^39^ Reads were de-multiplexed, then mapped to pCis plasmid sequence consisting of ITR-flanked transgene and plasmid backbone using the Burrows-Wheeler aligner-maximal exact match tool.^40^ Aligned reads were visualized with Integrated Genomics Viewer tool version 2.14.0 with soft clipping on.^41^

To analyze the ITR flip/flop configuration, four references from ITR to ITR representing each configuration were created in a fasta file (flip/flip, flip/flop, flop/flop, flop/flip). ITR flip orientation is defined as the B-arm being closest to the open end of the vector genome. The B-arm is defined as 5′-CGGGCGACCTTTGGTCGCCCG-3′ or the reverse complementary sequence. C-arm is defined as 5′-CGCCCGGGCAAAGC CCGGGCG-3′ or the reverse complementary sequence. Full-length reads were mapped to the fasta file reference. Reads aligning to each of the four flip/flop conformations were tabulated.

### RNA-seq

RNA-seq and analysis was carried out by Fornax Biotech (https://www.fornaxbio.com) under the vendor’s standard conditions. Briefly, mouse liver RNA was isolated using TRIzol reagent and subjected to rRNA depletion and fragmentation for RNA-seq library preparation. Libraries were sequenced on an Illumina NextSeq 500 with paired-end 150 bases in size with an average of 126.5 million reads in depth per liver sample. Reads were mapped to reference pCis plasmid sequence consisting of ITR-flanked transgene and plasmid backbone. In total, 20 mice in 4 treatment groups (n = 5 mice per group) were analyzed: empty rAAV9, scAAV9.EGFP generated using 100%, 10%, or 1% pCis.

### Statistical analysis

Data were presented as mean ± SD. Comparison among multiple groups was analyzed by one-way ANOVA followed by Dunnett’s multiple comparisons test. GraphPad Prism 10 was used for statistical analysis and data plotting.

## Data and code availability

PacBio and RNA-seq data can be found in the NCBI’s Sequence Read Archive (SRA) using accession number SUB13812505. All other data are provided in this paper.

## Supplemental information

Document S1. Figures S1–S8 and Tables S1 and S2

Document S2. Article plus supplemental information

## References

[1] Xiao X., Li J., Samulski R.J. (1998). Production of high-titer recombinant adeno-associated virus vectors in the absence of helper adenovirus. J. Virol..

[2] Cameau E., Pedregal A., Glover C. (2019). Cost modelling comparison of adherent multi-trays with suspension and fixed-bed bioreactors for the manufacturing of gene therapy products. Cell & Gene Therapy Insights.

[3] Guimaro M.C., Afione S.A., Tanaka T., Chiorini J.A. (2020). Rescue of Adeno-Associated Virus Production by shRNA Cotransfection. Hum. Gene Ther..

[4] Lu Y., He W., Huang X., He Y., Gou X., Liu X., Hu Z., Xu W., Rahman K., Li S. (2021). Strategies to package recombinant Adeno-Associated Virus expressing the N-terminal gasdermin domain for tumor treatment. Nat. Commun..

[5] Wright J.F. (2014). Product-Related Impurities in Clinical-Grade Recombinant AAV Vectors: Characterization and Risk Assessment. Biomedicines.

[6] Pupo A., Fernández A., Low S.H., François A., Suárez-Amarán L., Samulski R.J. (2022). AAV vectors: The Rubik's cube of human gene therapy. Mol. Ther..

[7] Smith J., Grieger J., Samulski R.J. (2018). Overcoming Bottlenecks in AAV Manufacturing for Gene Therapy. Cell Gene Therapy Insights.

[8] Pena A. (2018). https://musculardystrophynews.com/news/fda-places-clinical-hold-trial-sarepta-duchenne-gene-therapy/.

[9] Young C.M., Quinn C., Trusheim M.R. (2022). Durable cell and gene therapy potential patient and financial impact: US projections of product approvals, patients treated, and product revenues. Drug Discov. Today.

[10] Fu Q., Lee Y.S., Green E.A., Wang Y., Park S.Y., Polanco A., Lee K.H., Betenbaugh M., McNally D., Yoon S. (2023). Design space determination to optimize DNA complexation and full capsid formation in transient rAAV manufacturing. Biotechnol. Bioeng..

[11] Zhao H., Lee K.J., Daris M., Lin Y., Wolfe T., Sheng J., Plewa C., Wang S., Meisen W.H. (2020). Creation of a High-Yield AAV Vector Production Platform in Suspension Cells Using a Design-of-Experiment Approach. Mol. Ther. Methods Clin. Dev..

[12] Tang Q., Keeler A.M., Zhang S., Su Q., Lyu Z., Cheng Y., Gao G., Flotte T.R. (2020). Two-Plasmid Packaging System for Recombinant Adeno-Associated Virus. Biores. Open Access.

[13] van Lieshout L.P., Rubin M., Costa-Grant K., Ota S., Golebiowski D., Panico T., Wiberg E., Szymczak K., Gilmore R., Stanvick M. (2023). A novel dual-plasmid platform provides scalable transfection yielding improved productivity and packaging across multiple AAV serotypes and genomes. Mol. Ther. Methods Clin. Dev..

[14] Strobel B., Klauser B., Hartig J.S., Lamla T., Gantner F., Kreuz S. (2015). Riboswitch-mediated Attenuation of Transgene Cytotoxicity Increases Adeno-associated Virus Vector Yields in HEK-293 Cells. Mol. Ther..

[15] Schnödt M., Schmeer M., Kracher B., Krüsemann C., Espinosa L.E., Grünert A., Fuchsluger T., Rischmüller A., Schleef M., Büning H. (2016). DNA Minicircle Technology Improves Purity of Adeno-associated Viral Vector Preparations. Mol. Ther. Nucleic Acids.

[16] Karbowniczek K., Rothwell P., Extance J., Milsom S., Lukashchuk V., Bowes K., Smith D., Caproni L. (2017). Doggybone™ DNA: an advanced platform for AAV production. Cell Gene Therapy Insights.

[17] Wang J., Zhang Y., Mendonca C.A., Yukselen O., Muneeruddin K., Ren L., Liang J., Zhou C., Xie J., Li J. (2022). AAV-delivered suppressor tRNA overcomes a nonsense mutation in mice. Nature.

[18] Schmit P.F., Pacouret S., Zinn E., Telford E., Nicolaou F., Broucque F., Andres-Mateos E., Xiao R., Penaud-Budloo M., Bouzelha M. (2020). Cross-Packaging and Capsid Mosaic Formation in Multiplexed AAV Libraries. Mol. Ther. Methods Clin. Dev..

[19] Nonnenmacher M., van Bakel H., Hajjar R.J., Weber T. (2015). High capsid-genome correlation facilitates creation of AAV libraries for directed evolution. Mol. Ther..

[20] Wong L.C., Singh S., Wang H.P., Hsu C.J., Hu S.C., Lee W.T. (2019). FOXG1-Related Syndrome: From Clinical to Molecular Genetics and Pathogenic Mechanisms. Int. J. Mol. Sci..

[21] Chen L., Liu Y. (2023). Clinical and genetic characteristics of children with COX20-associated mitochondrial disorder: case report and literature review. BMC Med. Genom..

[22] Lahey H.G., Webber C.J., Golebiowski D., Izzo C.M., Horn E., Taghian T., Rodriguez P., Batista A.R., Ellis L.E., Hwang M. (2020). Pronounced Therapeutic Benefit of a Single Bidirectional AAV Vector Administered Systemically in Sandhoff Mice. Mol. Ther..

[23] Gonçalves M.A.F.V. (2005). Adeno-associated virus: from defective virus to effective vector. Virol. J..

[24] Ward P., Elias P., Linden R.M. (2003). Rescue of the adeno-associated virus genome from a plasmid vector: evidence for rescue by replication. J. Virol..

[25] Wang X.S., Ponnazhagan S., Srivastava A. (1996). Rescue and replication of adeno-associated virus type 2 as well as vector DNA sequences from recombinant plasmids containing deletions in the viral inverted terminal repeats: selective encapsidation of viral genomes in progeny virions. J. Virol..

[26] Samulski R.J., Srivastava A., Berns K.I., Muzyczka N. (1983). Rescue of adeno-associated virus from recombinant plasmids: gene correction within the terminal repeats of AAV. Cell.

[27] Tai P.W.L., Xie J., Fong K., Seetin M., Heiner C., Su Q., Weiand M., Wilmot D., Zapp M.L., Gao G. (2018). Adeno-associated Virus Genome Population Sequencing Achieves Full Vector Genome Resolution and Reveals Human-Vector Chimeras. Mol. Ther. Methods Clin. Dev..

[28] Chadeuf G., Ciron C., Moullier P., Salvetti A. (2005). Evidence for encapsidation of prokaryotic sequences during recombinant adeno-associated virus production and their in vivo persistence after vector delivery. Mol. Ther..

[29] Earley L.F., Conatser L.M., Lue V.M., Dobbins A.L., Li C., Hirsch M.L., Samulski R.J. (2020). Adeno-Associated Virus Serotype-Specific Inverted Terminal Repeat Sequence Role in Vector Transgene Expression. Hum. Gene Ther..

[30] Haberman R.P., McCown T.J., Samulski R.J. (2000). Novel transcriptional regulatory signals in the adeno-associated virus terminal repeat A/D junction element. J. Virol..

[31] Yang J.L., Zhang L.P., Xu J., Ma P.M., Yang R.J., Jia G.D., Shen W.R., Yang X.L. (2023). Novel Repression System for Gene Expression Regulation during Recombinant Adeno-Assoiciated Virus and Lentiviral Vector Manufacturing. Mol. Ther..

[32] Tran N.T., Lecomte E., Saleun S., Namkung S., Robin C., Weber K., Devine E., Blouin V., Adjali O., Ayuso E. (2022). Human and Insect Cell-Produced Recombinant Adeno-Associated Viruses Show Differences in Genome Heterogeneity. Hum. Gene Ther..

[33] Giles A., Lock M., Chen S.J., Turner K.B., Wesolowski G., Prongay A., Petkov B.N., Olagbegi K., Yan H., Wilson J.M. (2023). Significant differences in capsid properties and potency between AAV vectors produced in Sf9 and HEK293 cells. Hum. Gene Ther..

[34] Su W., Patrício M.I., Duffy M.R., Krakowiak J.M., Seymour L.W., Cawood R. (2022). Self-attenuating adenovirus enables production of recombinant adeno-associated virus for high manufacturing yield without contamination. Nat. Commun..

[35] Ronzitti G., Gross D.A., Mingozzi F. (2020). Human Immune Responses to Adeno-Associated Virus (AAV) Vectors. Front. Immunol..

[36] Su Q., Sena-Esteves M., Gao G. (2020). Analysis of Recombinant Adeno-Associated Virus (rAAV) Purity Using Silver-Stained SDS-PAGE. Cold Spring Harb. Protoc..

[37] Ai J., Ibraheim R., Tai P.W.L., Gao G. (2017). A Scalable and Accurate Method for Quantifying Vector Genomes of Recombinant Adeno-Associated Viruses in Crude Lysate. Hum. Gene Ther. Methods.

[38] Crowe A.R., Yue W. (2019). Semi-quantitative Determination of Protein Expression using Immunohistochemistry Staining and Analysis: An Integrated Protocol. Bio. Protoc..

[39] Afgan E., Sloggett C., Goonasekera N., Makunin I., Benson D., Crowe M., Gladman S., Kowsar Y., Pheasant M., Horst R., Lonie A. (2015). Genomics Virtual Laboratory: A Practical Bioinformatics Workbench for the Cloud. PLoS One.

[40] Li H. (2013). Aligning sequence reads, clone sequences and assembly contigs with BWA-MEM. arXiv.

[41] Robinson J.T., Thorvaldsdóttir H., Winckler W., Guttman M., Lander E.S., Getz G., Mesirov J.P. (2011). Integrative genomics viewer. Nat. Biotechnol..

