# Supplementary material for: Producing high-quantity and high-quality recombinant adeno-associated virus by low-cis triple transfection
Source: Mol Ther Methods Clin Dev. 2024 Mar 12;32(2):101230. doi: 10.1016/j.omtm.2024.101230 (PMC10979107; doi:10.1016/j.omtm.2024.101230)
Supplement: Document S1. Figures S1–S8 and Tables S1 and S2 [file mmc1.pdf]

OMTM, Volume *32*

Supplemental information

Producing high-quantity and high-quality recombinant adeno-associated virus by low-cis triple transfection

Hao Liu, Yue Zhang, Mitchell Yip, Lingzhi Ren, Jialing Liang, Xiupeng Chen, Nan Liu, Ailing Du, Jiaming Wang, Hao Chang, Hyejin Oh, Chen Zhou, Ruxiao Xing, Mengyao Xu, Peiyi Guo, Dominic Gessler, Jun Xie, Phillip W.L. Tai, Guangping Gao, and Dan Wang

Table S1. Plasmid usage in different scales, cell lines and triple transfection methods.

HEK293 cells	Culture vessel	Scale	Experiment	Plasmid	Standard triple transfection	Low-cis triple transfection	
					100% pCis	10% pCis	1% pCis
Adherent	12-well plate	1 well (5×10^5 cells)	Figures 1B, C, D; 2A; S1; S2; S4B	pCis<br>pTrans<br>pHelper	0.5 µg<br>0.5 µg<br>0.5 µg	0.05 µg<br>0.5 µg<br>0.5 µg	0.005 µg<br>0.5 µg<br>0.5 µg
Adherent	Roller bottle	10 roller bottles (1×10^9 cells)	Figures 1E, F; 2B-D; S3B; S4B; S6A	pCis<br>pTrans<br>pHelper	1.5 mg<br>1.5 mg<br>1.5 mg	0.15 mg<br>1.5 mg<br>1.5 mg	0.015 mg<br>1.5 mg<br>1.5 mg
Suspension	14-mL tube	3 mL culture (3×10^6 cells)	Figures 5A, B, D	pCis<br>pTrans<br>pHelper	1 µg<br>1 µg<br>1 µg	0.1 µg<br>1 µg<br>1 µg	0.01 µg<br>1 µg<br>1 µg
Suspension	125-mL shaker flask	30 mL culture (3×10^7 cells)	Figures 5B, C, D	pCis<br>pTrans<br>pHelper	10 µg<br>10 µg<br>10 µg	1 µg<br>10 µg<br>10 µg	0.1 µg<br>10 µg<br>10 µg

Table S2. PacBio sequencing analysis of encapsidated host cell genome and pHelper plasmid.

pCis input	Total reads #	Host cell genome reads #	Host cell genome ratio	pHelper reads #	pHelper ratio	Host cell genome + pHelper ratio
100% pCis	201519	3587	1.78%	817	0.41%	2.19%
10% pCis	264199	3211	1.22%	1850	0.70%	1.92%
1% pCis	210617	3006	1.43%	1685	0.80%	2.23%

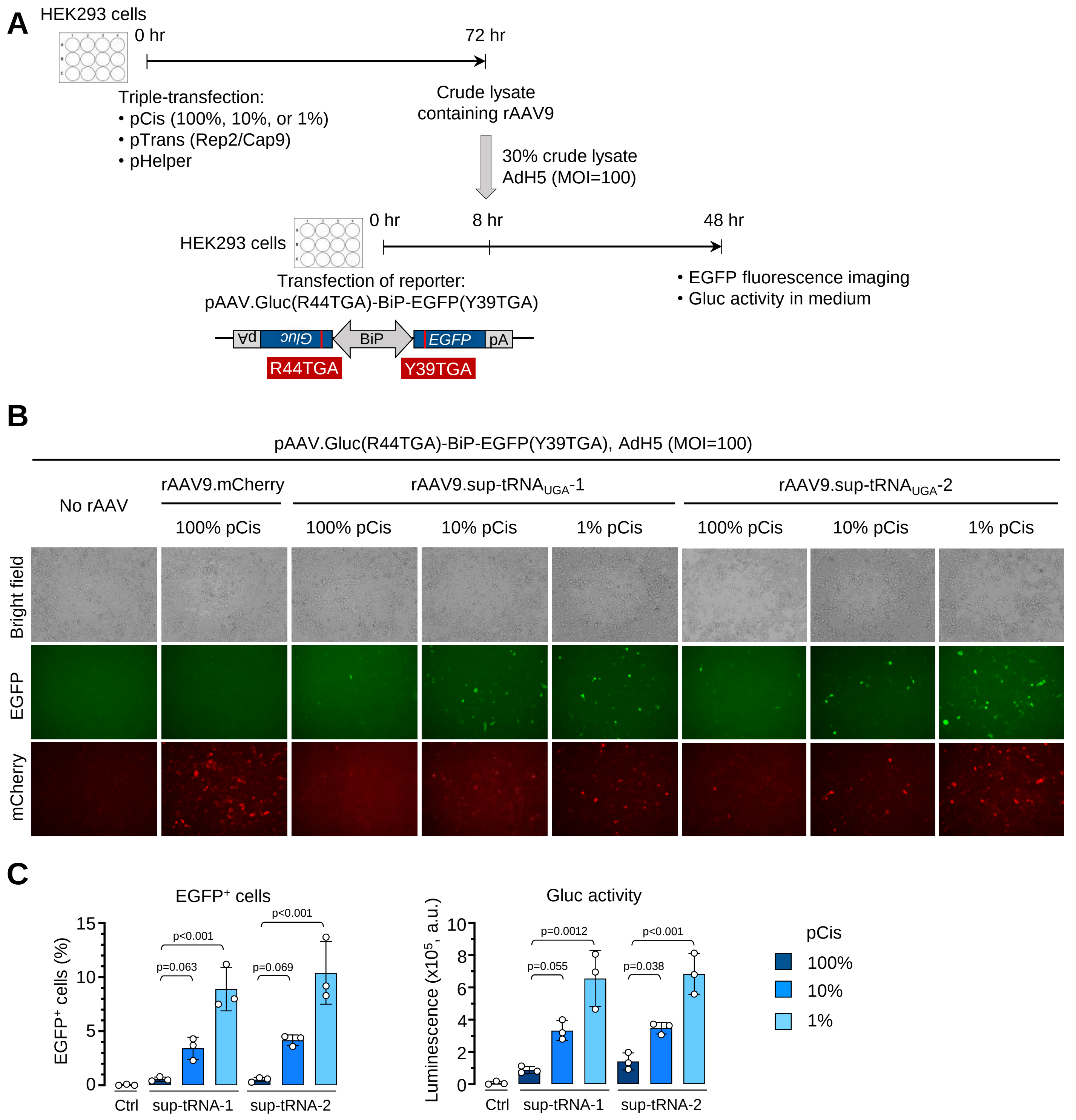

Figure S1. ssAAV9.sup-tRNA$_{UGA}$ generated by low-cis triple transfection is functional.

A, Schematic diagram showing the workflow to test UGA readthrough capability of ssAAV9.sup-tRNA$_{UGA}$ produced by standard or low-cis triple transfection. The readthrough reporter construct contains mutant Gaussia luciferase (*Gluc*) and *EGFP* genes (blue boxes) each bearing a TGA nonsense mutation (red lines and boxes), driven by a bi-directional promoter (BiP). AdH5: human adenovirus serotype 5 (to enhance rAAV transduction). MOI: multiplicity of infection. **B**, Representative images showing EGFP fluorescence due to UGA readthrough and mCherry fluorescence due to vector transduction. **C**, Quantification of EGFP-positive cells and Gluc activity in culture medium. More details of quantification procedures are described in Methods. In (**C**), data are mean ± s.d. of three biological replicates. Statistical analysis was performed using one-way analysis of variance (ANOVA) followed by Dunnett's multiple comparisons test against 100% pCis group.

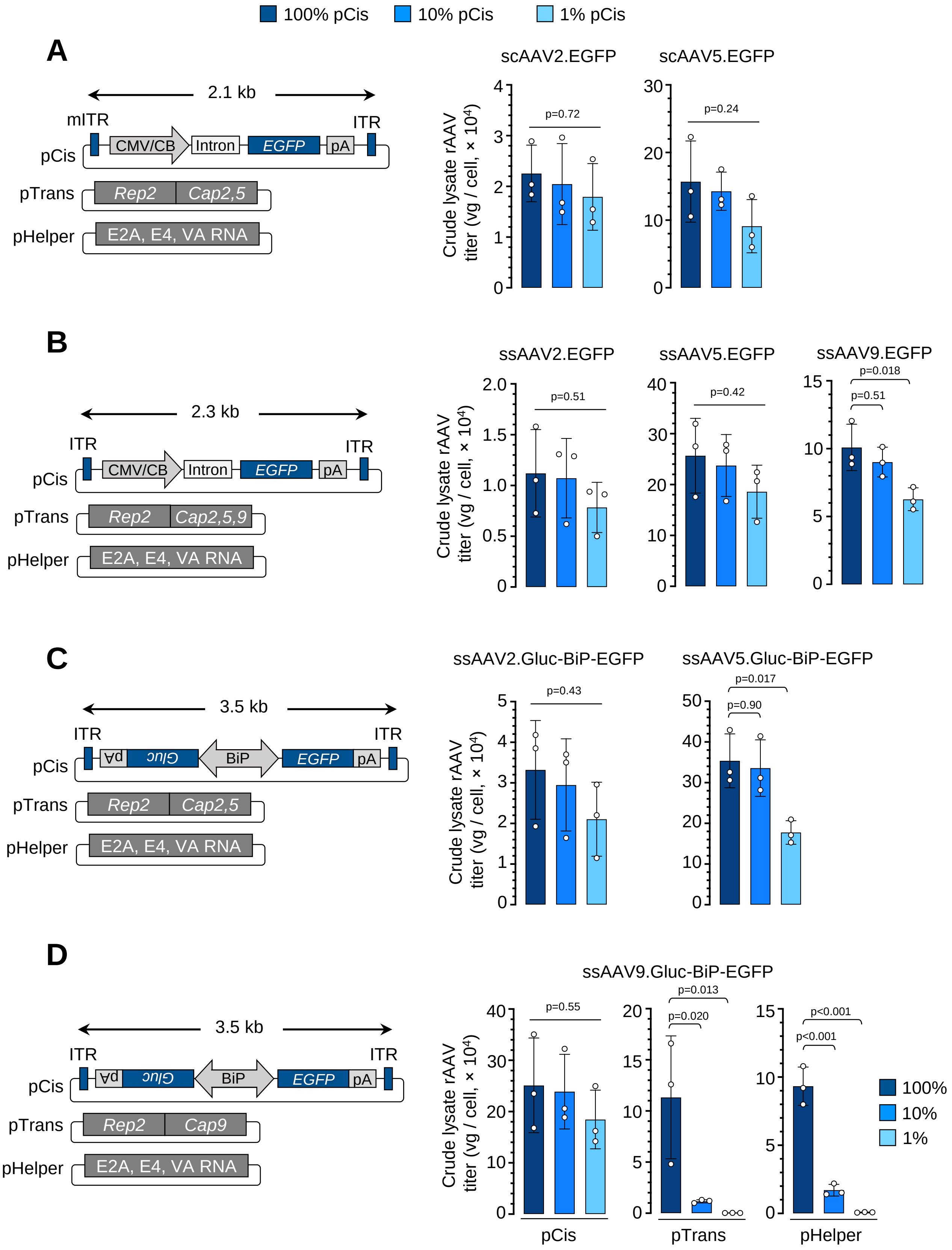
100% pCis
10% pCis
1% pCis
A
2.1 kb
mITR
ITR
pCis
CMV/CB
Intron
EGFP
pA
pTrans
Rep2
Cap2,5
pHelper
E2A, E4, VA RNA
scAAV2.EGFP
scAAV5.EGFP
Crude lysate rAAV titer (vg / cell, × 10^4)
p=0.72
p=0.24
B
2.3 kb
ITR
Cap2,5,9
ssAAV2.EGFP
ssAAV5.EGFP
ssAAV9.EGFP
p=0.51
p=0.42
p=0.018
C
3.5 kb
Gluc
BiP
ssAAV2.Gluc-BiP-EGFP
ssAAV5.Gluc-BiP-EGFP
p=0.43
p=0.017
p=0.90
D
Cap9
ssAAV9.Gluc-BiP-EGFP
p=0.55
p=0.013
p=0.020
p<0.001
100%
10%
1%
pCis
pTrans
pHelper

Figure S2. Low-cis triple transfection is broadly applicable.

A, Left panel: self-complementary vector genome structure of pCis that expresses EGFP by CB6 promoter, pTrans that expresses Rep2/Cap2 or Rep2/Cap5, and pHelper. Right panel: Packaging yield of scAAV2.EGFP and scAAV5.EGFP using various amounts of pCis in small-scale rAAV production assay. Detailed plasmid usage is shown in Supplementary Table 1. mITR: mutant ITR for generating self-complementary rAAV. **B**, Left panel: vector genome structure of pCis that expresses EGFP by CB6 promoter, pTrans that expresses Rep2/Cap2 or Rep2/Cap5 or Rep2/Cap9, and pHelper. Right panel: Packaging yield of ssAAV2.EGFP, ssAAV5.EGFP and ssAAV9.EGFP using various amounts of pCis in small-scale rAAV production assay. **C**, Left panel: vector genome structure of pCis that expresses Gluc and EGFP by a bi-directional promoter (BiP), pTrans that expresses Rep2/Cap2 or Rep2/Cap5, and pHelper. Right panel: Packaging yield of ssAAV2.Gluc-BiP-EGFP and ssAAV5.Gluc-Bip-EGFP using various amounts of pCis in small-scale rAAV production assay. **D,** Left panel: vector genome structure of pCis that expresses Gluc and EGFP by a bi-directional promoter (BiP), pTrans that expresses Rep2/Cap9 and pHelper. Right panel: Packaging yield of ssAAV9.Gluc-BiP-EGFP using various amounts of pCis, pTrans or pHelper in small-scale rAAV production assay. sc: self-complementary; ss: single-stranded. In (**A-D**), data are mean ± s.d. of three biological replicates. Statistical analysis was performed using one-way ANOVA followed by Dunnett's multiple comparisons test against 100% pCis group.

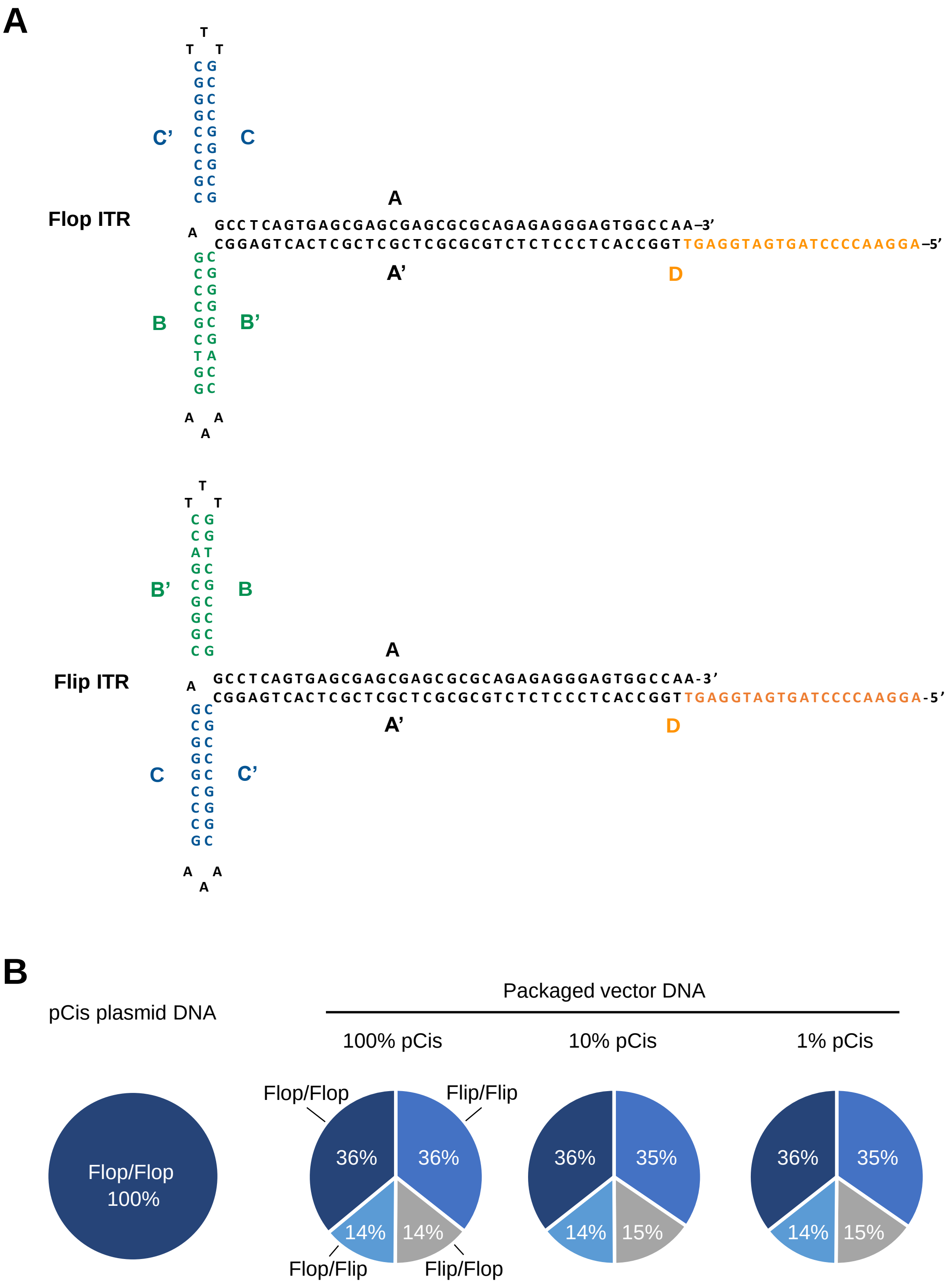

Figure S3. Diversified ITR configuration in packaged vector DNA.

A, ITR configuration is defined as Flop (upper) when C arm is closer to the open end, or Flip (lower) when B arm is closer to the open end. **B**, Pie charts showing ITR configuration distribution in pCis plasmid (pAAVsc.EGFP) or packaged vector DNA (scAAV9.EGFP) produced using different pCis input as described in Figure 2B. Note that two ITRs in one vector DNA create four combinations of configuration.

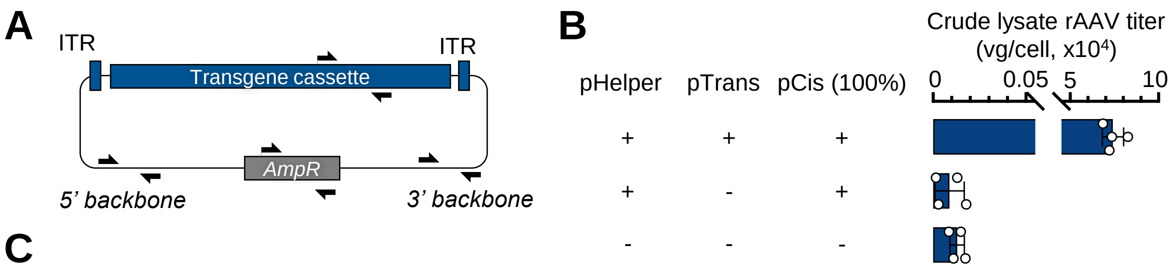

C

Scale	rAAV (source)	Biological replicates		100% pCis	10% pCis	1% pCis
10 roller bottles	scAAV9. EGFP, #1 (CsCl purified)	1	5' backbone	10	7.1	4.9
			3' backbone	5.6	3.1	1.9
			AmpR	10	6.2	4.0
10 roller bottles	scAAV9. EGFP, #2 (CsCl purified)	1	5' backbone	10	3.0	2.4
			3' backbone	5.1	1.3	0.70
			AmpR	7.9	2.6	2.2
10 roller bottles	ssAAV9. Gluc-BiP-EGFP (CsCl purified)	1	5' backbone	3.0	n/a	0.70
			3' backbone	1.9	n/a	0.50
			AmpR	3.7	n/a	1.0
10 roller bottles	ssAAV5. Gluc-BiP-EGFP (CsCl purified)	1	5' backbone	14	8.8	6.7
			3' backbone	12	7.4	6.3
			AmpR	13	8.6	7.4
12-well plate	scAAV8.EGFP (crude lysate)	3	5' backbone	7.1	2.4	1.1
			3' backbone	3.4	1.0	0.50
			AmpR	6.4	2.6	1.3
12-well plate	scAAV9.EGFP (crude lysate)	3	5' backbone	8.1	2.5	2.4
			3' backbone	5.3	1.1	0.70
			AmpR	6.4	2.4	2.8
12-well plate	ssAAV9.EGFP (crude lysate)	3	5' backbone	2.0	0.38	0.36
			3' backbone	1.3	0.22	0.11
			AmpR	2.0	0.38	0.36
12-well plate	ssAAV5.EGFP (crude lysate)	3	*AmpR*	6.6	2.1	2.1
12-well plate	ssAAV2.EGFP (crude lysate)	3	*AmpR*	4.0	1.4	1.6

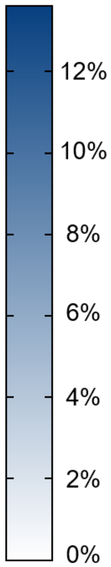

Figure S4. Low-cis triple transfection produces high-quality AAV vectors with reduced backbone DNA encapsidation.

A, Schematic of the pCis showing Taqman probe designs that target transgene, 5' backbone, ampicillin resistant gene (*AmpR*), or 3' backbone. **B**, Bar graph showing the crude lysate rAAV titers under transfection conditions that included various plasmid combinations. Note that when pHelper and pCis (pAAV.EGFP) were transfected without pTrans (hence no formation of AAV capsid), *EGFP* was at background level in crude lysate as determined by ddPCR, demonstrating that DNase I treatment of crude lysate effectively removed plasmid carryover (also see Materials and Methods). **C,** Heat map showing the percentage of pCis plasmid backbone DNA normalized to transgene in AAV vectors differing in production scale, capsid serotype, source of material, transgene, and genome configuration as indicated. Cleared crude lysates or purified rAAV was treated with DNase-I and protease K, followed by duplex ddPCR with one probe targeting transgene, the other targeting 5', 3' or AmpR backbone. For assays involving multiple biological replicates, the average value was shown. sc: self-complementary; ss: single-stranded; n/a: not applicable (rAAV production was not performed).

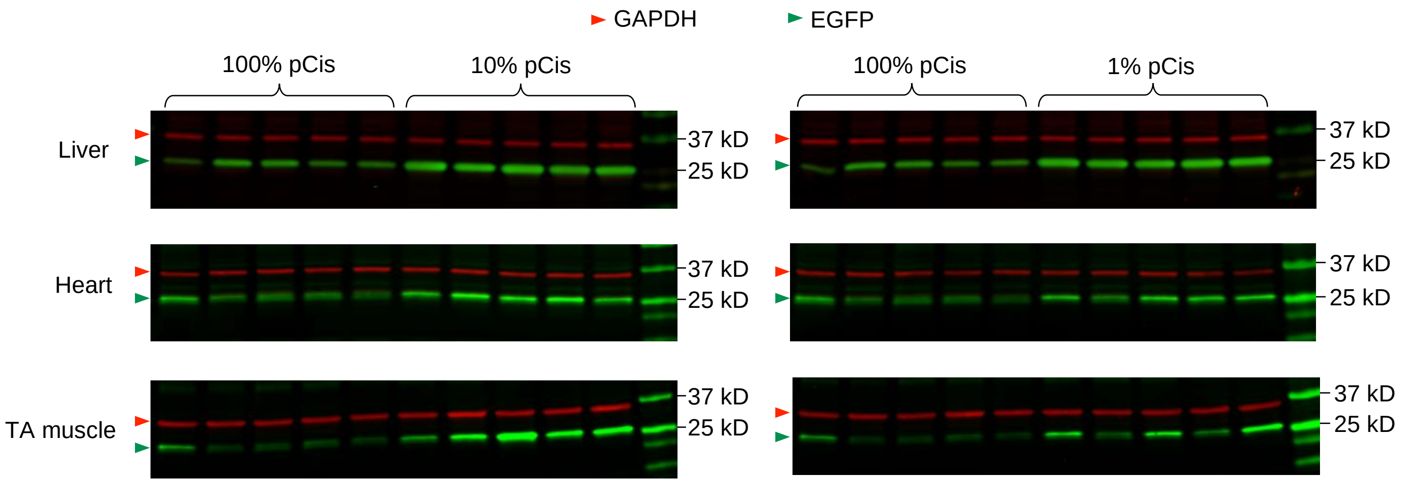

Figure S5. Original western blot images to characterize scAAV9.EGFP *in vivo* potency.

Western blotting images showing EGFP protein expression (green arrowheads) and GAPDH protein expression (red arrowheads, as loading control) in various tissues from mice as described in Figure 3A.

.

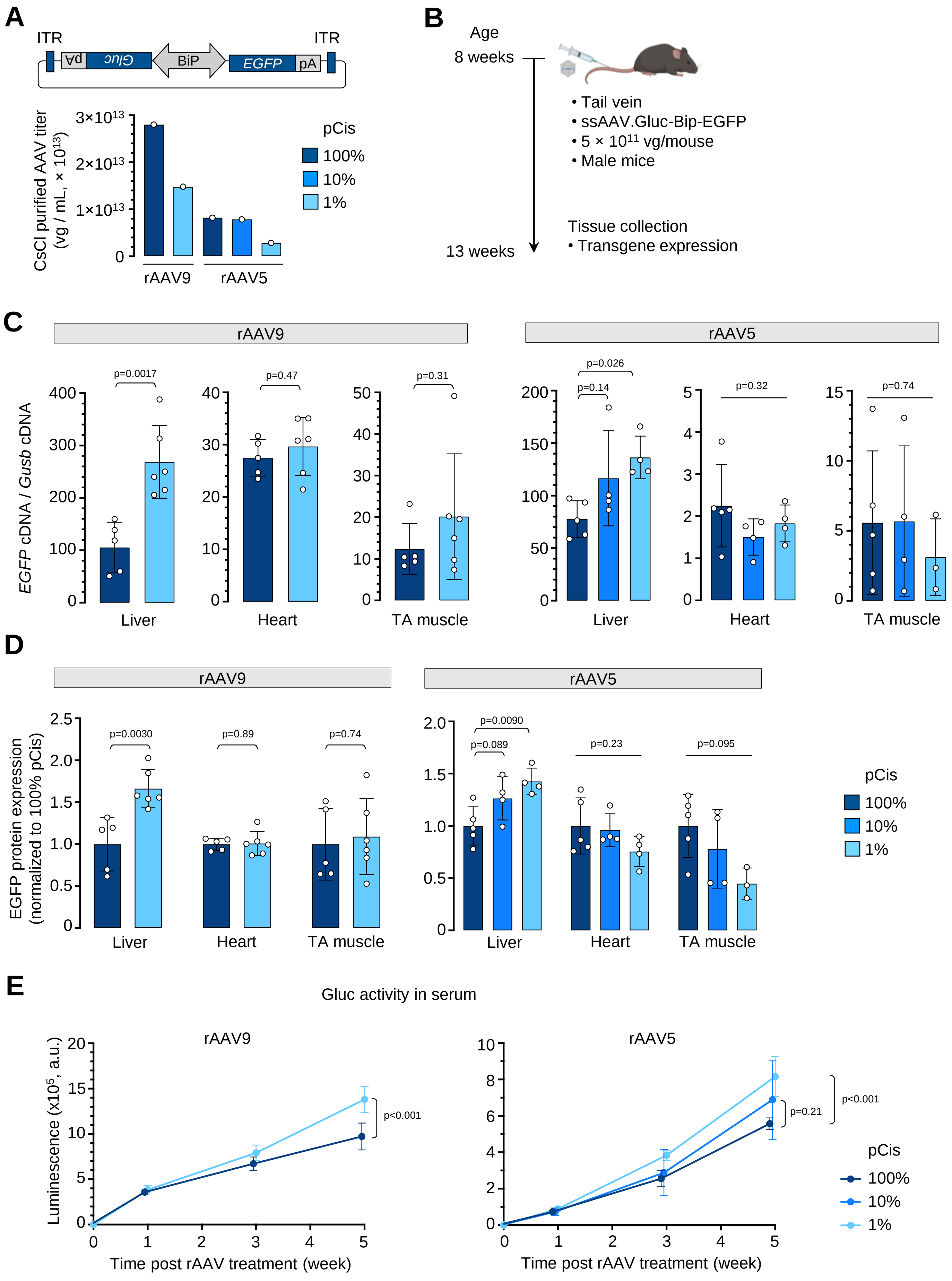
A
ITR
ITR
pA
Gluc
BiP
EGFP
pA
CsCl purified AAV titer (vg / mL, × 10^13)
pCis
100%
10%
1%
rAAV9
rAAV5
B
Age
8 weeks
• Tail vein
• ssAAV.Gluc-Bip-EGFP
• 5 × 10^11 vg/mouse
• Male mice
Tissue collection
• Transgene expression
13 weeks
C
rAAV9
rAAV5
EGFP cDNA / Gusb cDNA
p=0.0017
p=0.47
p=0.31
p=0.026
p=0.14
p=0.32
p=0.74
Liver
Heart
TA muscle
D
rAAV9
rAAV5
EGFP protein expression (normalized to 100% pCis)
p=0.0030
p=0.89
p=0.74
p=0.0090
p=0.089
p=0.23
p=0.095
Liver
Heart
TA muscle
pCis
100%
10%
1%
E
Gluc activity in serum
rAAV9
rAAV5
Luminescence (x10^5, a.u.)
p<0.001
p=0.21
p<0.001
Time post rAAV treatment (week)
pCis
100%
10%
1%

Figure S6. Extension of low-cis triple transfection to produce rAAVs of different transgenes and serotypes for *in vivo* administration.

A, Upper panel: vector genome structure of pCis that expresses *Gluc* and *EGFP* by a bi-directional promoter (BiP). Lower panel: packaging yield of ssAAV9.Gluc-BiP-EGFP and ssAAV5.Gluc-BiP-EGFP using different pCis input in large-scale rAAV production. **B**, Schematic diagram showing workflow. **C-D,** Quantification of *EGFP* cDNA (**C**) and protein (**D**) levels in the liver, heart and TA muscle from mice as described in (**B**). The original western blotting images for (**D**) are shown in Supplementary Figure 7. **E**, Gluc activity in mouse sera collected at different time points post rAAV treatment. Statistical analysis was performed using two-way ANOVA (for rAAV9), or two-way ANOVA followed by Dunnett's multiple comparisons test against 100% pCis group (for rAAV5). In (**C-D**), data are mean ± s.d. of individual animals (circles). Statistical analysis was performed using one-way ANOVA followed by Dunnett's multiple comparisons test against 100% pCis group.

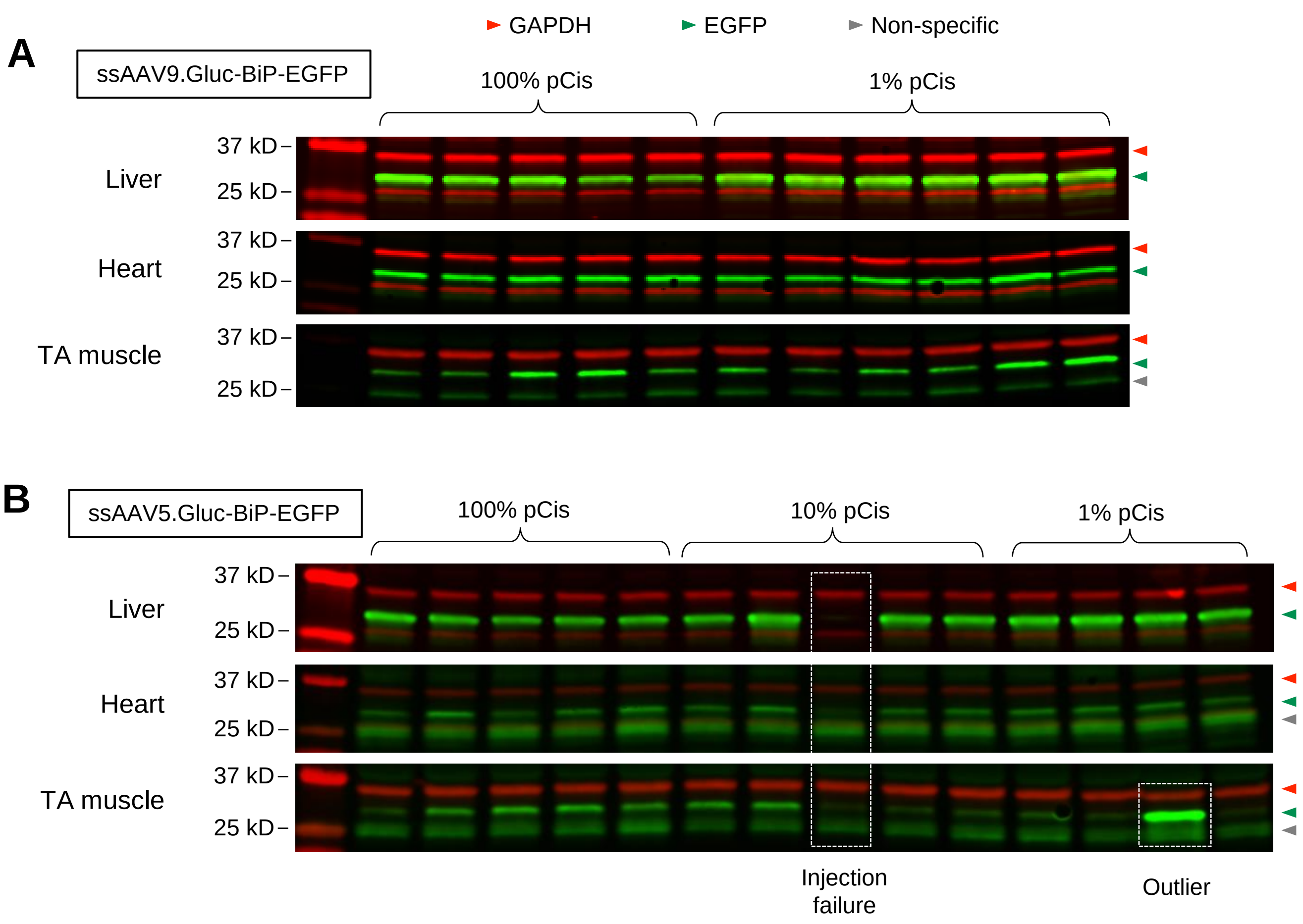

Figure S7. Original western blot images to characterize ssAAV9.Gluc-BiP-EGFP and ssAAV5.Gluc-BiP-EGFP *in vivo* potency.

A, Western blotting images showing EGFP protein expression (green arrowheads) and GAPDH protein expression (red arrowheads, as loading control) in various tissues from mice treated with ssAAV9.Gluc-BiP-EGFP as described in Supplementary Figure 6B. Gray arrowheads indicate non-specific bands. **B**, Western blotting images showing EGFP protein expression (green arrowheads) and GAPDH protein expression (red arrowheads, as loading control) in various tissues from mice treated with ssAAV5.Gluc-BiP-EGFP as described in Supplementary Figure 6B. Gray arrowheads indicate non-specific bands.

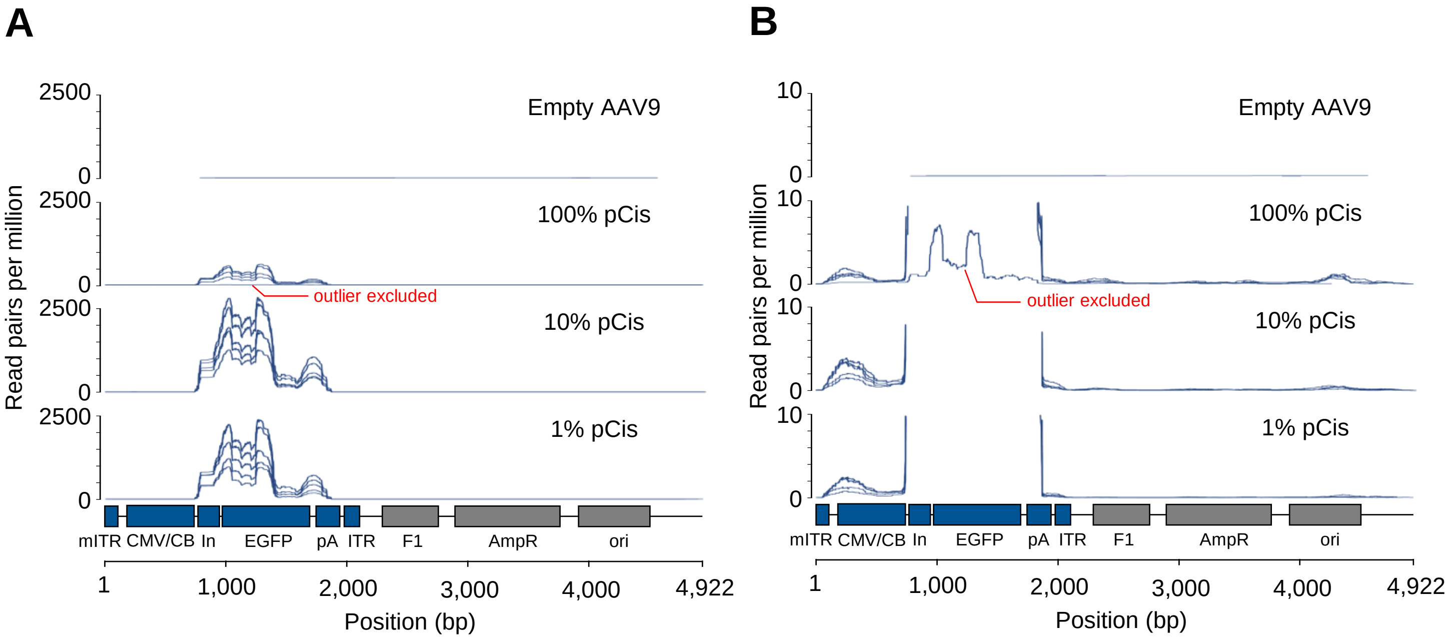

Figure S8. RNA sequencing results of all samples.

A-B, Coverage plots of RNA sequencing reads mapped to the ITR-flanked scAAV9.EGFP vector genome (blue) or pCis backbone DNA (gray) as described in Figure 4E, but results of all liver samples (n=5 mice per group) are included. Note that the Y-axis scales are different between (**A**) and (**B**). One sample in the 100% pCis group (outlier excluded) contained very low number of mapped reads compared with the other four samples in the same group, and therefore was excluded in the analysis presented in Figure 4E and 4F.
